# APP promotes osteoblast survival and bone formation by regulating mitochondrial function and preventing oxidative stress

**DOI:** 10.1038/s41419-018-1123-7

**Published:** 2018-10-22

**Authors:** Jin-Xiu Pan, Fulei Tang, Fei Xiong, Lei Xiong, Peng Zeng, Bo Wang, Kai Zhao, Haohan Guo, Cui Shun, Wen-Fang Xia, Lin Mei, Wen-Cheng Xiong

**Affiliations:** 1 Department of Neuroscience, Case Western Reserve University, Cleveland, OH 44106 USA; 2 Department of Neuroscience and Regenerative Medicine, Medical College of Georgia, Augusta University, Augusta, GA 30912 USA; 3 Charlie Norwood VA Medical Center, Augusta, GA 30912 USA; 4 Louis Stokes Cleveland VAMC, Cleveland, OH 44106 USA; 5 The Center for Biomedical Research, Tongji Hospital, Tongji Medical College, Huazhong University of Science and Technology, 430030 Wuhan, China; 6 Department of Rheumatology, Union Hospital, Tongji Medical College, Huazhong University of Science and Technology, 430022 Wuhan, China

## Abstract

Amyloid precursor protein (APP) is ubiquitously expressed in various types of cells including bone cells. Mutations in App gene result in early-onset Alzheimer’s disease (AD). However, little is known about its physiological function in bone homeostasis. Here, we provide evidence for APP’s role in promoting bone formation. Mice that knocked out App gene (APP^−/−^) exhibit osteoporotic-like deficit, including reduced trabecular and cortical bone mass. Such a deficit is likely due in large to a decrease in osteoblast (OB)-mediated bone formation, as little change in bone resorption was detected in the mutant mice. Further mechanical studies of APP^−/−^ OBs showed an impairment in mitochondrial function, accompanied with increased reactive oxygen species (ROS) and apoptosis. Intriguingly, these deficits, resemble to those in Tg2576 animal model of AD that expresses Swedish mutant APP (APPswe), were diminished by treatment with an anti-oxidant NAC (n-acetyl-l-cysteine), uncovering ROS as a critical underlying mechanism. Taken together, these results identify an unrecognized physiological function of APP in promoting OB survival and bone formation, implicate APPswe acting as a dominant negative factor, and reveal a potential clinical value of NAC in treatment of AD-associated osteoporotic deficits.

## Introduction

Alzheimer’s disease (AD) is the most common form of dementia, affecting 10% of people over 65 years old^1^. Osteoporosis is characterized by low bone mineral density (BMD) and micro-architectural deterioration of bone tissue. AD and osteoporosis are largely seen as independent disorders; however, a positive association of AD with reduced radiographic BMD^2,3^ and an increase in bone resorption^4^ have been reported. Multiple risk genes/loci identified in AD patients encode proteins critical for bone homeostasis, such as ApoEε4, TREM2, CD33, PYK2, VPS35, and SorL1^5–7^. A degree of comorbidity of both AD and osteoporosis is also supported by epidemiological studies. Both are multifactorial and polygenetic diseases, involving aging, environmental factors, chronic inflammation, and oxidative stress as their pathogenic mechanisms. However, it remains open if and how AD is linked with osteoporosis.

App, a mendelian gene for early-onset AD (EOAD), encodes a transmembrane protein, which can be cleaved by three proteases (α, β, and γ−secretases)^8–10^. Mutations in App gene identified in EOAD patients (e.g., Swedish mutation, APPswe) promote Aβ generation^11–13^. Is APPswe a risk factor for AD-associated bone loss or osteoporosis? In our previous publications^14,15^, we have employed two transgenic mouse models, Tg2576 and TgAPPswe-Ocn, to address this question. Tg2576 expresses APPswe ubiquitously under the control of prion promotor^14^, and TgAPPswe-Ocn selectively expresses APPswe in OB-lineage cells, which is under the control of osteocalcin (Ocn) promotor driven-Cre^15^. Both mouse models show osteoporotic deficits and impaired OB differentiation and function, suggesting a cell autonomous and deleterious effect of APPswe on OB differentiation, and raising a question about APP’s physiological role in this event.

Here, we determined APP’s physiological function in bone homeostasis by use of APP knock out (APP^−/−^) mice^16^. Similar osteoporotic deficits as those of Tg2576 and TgAPPswe-Ocn mice were detected. Further cellular mechanical studies suggest a critical role of APP in promoting OB mitochondrial function, and preventing cytochrome C release, reactive oxygen species (ROS) production, and OB cell death. Intriguingly, as that in Tg2576 mice, the deficits in APP^−/−^ mice were diminished by treatment with NAC (n-acetyl-l-cysteine), an anti-oxidant, implicating ROS as a denominator for skeleton deficits in both Tg2576 and APP^−/−^ mice. These results uncover a physiological role of APP in promoting OB survival, bone formation, and bone homeostasis, implicate a dominant negative role of APPswe in this event, and affirm NAC’s potential clinical value in the treatment of AD-associated osteoporotic disorders.

## Results

### Osteoporotic deficits in APP^−/−^ mice

APP^−/−^ mice appeared to be smaller in body size, with a reduced body weight (Fig. S1a–b). APP was detected not only in mouse brain, but also in bone marrow cells (BMSCs and BMMs), which was abolished in cells from APP^−/−^ mice (Fig. S1c). These results confirmed APP’s expression in bone cells as previous reports^14,15^, and verified APP^−/−^ mouse identity.

**Fig. 1:**
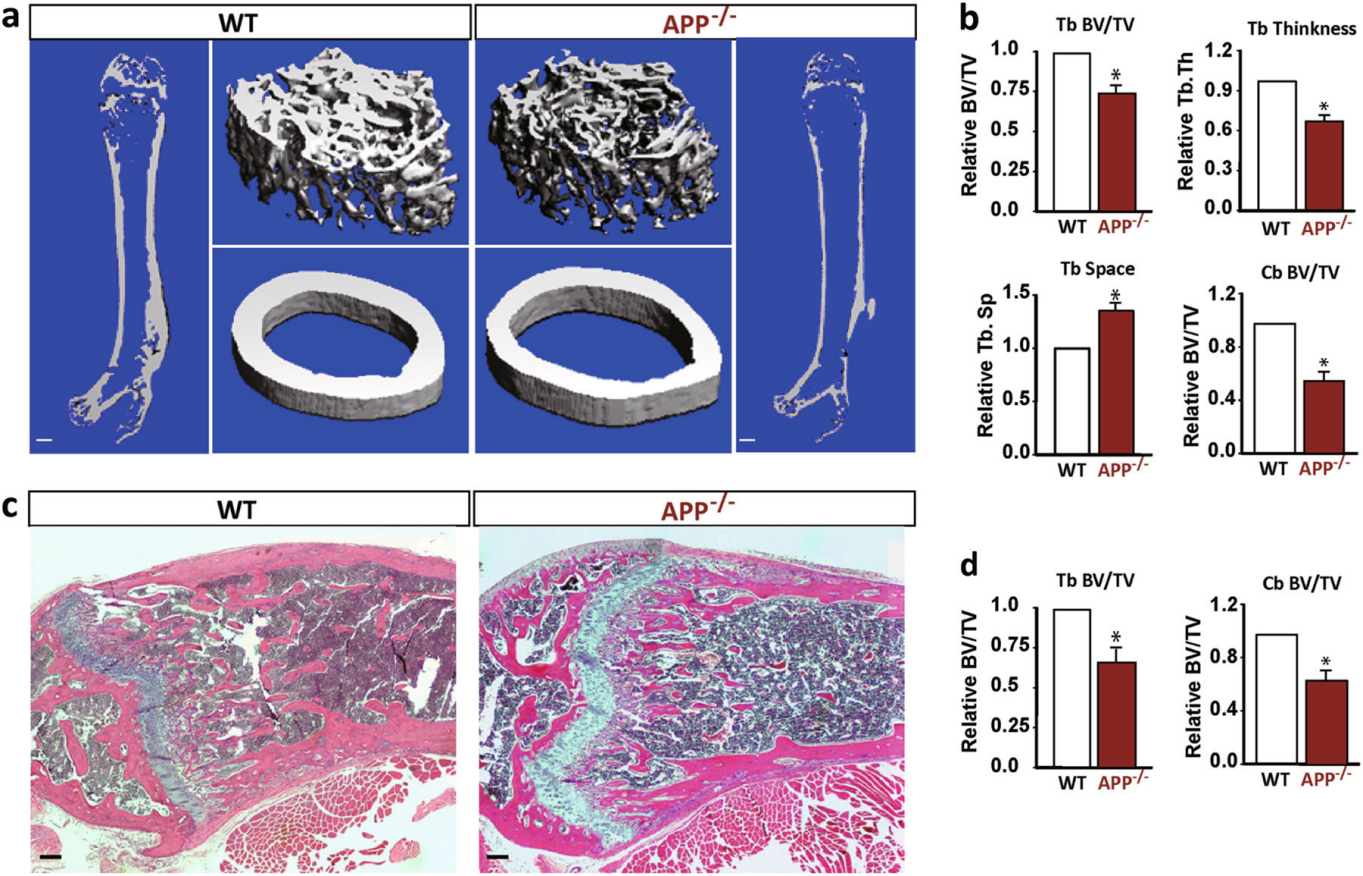
Loss of trabecular and cortical bone mass in APP^−/−^ femurs. Five different mice (2-month old, males) per genotype per assay were examined blindly. Female mice were also characterized, and similar phenotypes as those of male mutant mice were detected, but these data were not included here. **a**, **b** The μCT analysis of femurs from 2-M-old APP^−/−^ and WT mice. Representative 3-D images were shown in (a), scale bar, 1 mm. Quantification analyses (mean ± SEM, *n* = 5) were presented in **b**. *p *<* 0.05. Note that the trabecular bone (Tb) and cortical bone (Cb) volumes over total volumes (BV/TV), trabecular thickness (Tb, Th) and trabecular separation (Tb.Sp) were all deficient in APP^−/−^ femurs as compared with WT controls. **c**, **d** H & E staining analysis of femurs from 2-M old WT and APP^−/−^ mice. Representative images were shown in **c**. Scale bar, 100 μm. Quantification analyses (mean ± SEM, *n* = 5) were presented in **d**. *p < 0.05

We then compared the lone bone (femur) mass between WT and APP^−/−^ mice by microCT (μCT) analysis. Reductions in both trabecular and cortical bone volumes over total volumes were detected in APP^−/−^ mice, as compared with that of WT controls (Fig. 1a, b). Further analysis revealed decreased trabecular thickness (Tb. Th.) and increased trabecular space (Tb. Sp.) in APP^−/−^ mice (Fig. 1b). The reduced trabecular and cortical bone volumes in APP^−/−^ mice were further confirmed by H & E histological analysis (Fig. 1c, d and data not shown). These results demonstrate an osteoporotic deficit in APP^−/−^ mice, which resemble to that of Tg2576 mice^14,15^, revealing a role of APP in maintaining bone homeostasis.

### Increase of bone resorption in neonatal, but not adult, APP^−/−^ mice

To understand how APP regulates bone homeostasis, we first examined OC-mediated bone resorption, a critical event for bone homeostasis^17^, in 1-M and 2-M old WT and APP^−/−^ mice. Measuring serum levels of PYD (pyridinoline) (a marker for bone resorption)^18^ showed little difference, if there is any, between WT and APP^−/−^ mice at age of 2-M or older (Fig. S2a and data not shown). But, at age of 1-M old, an elevated levels of serum PYD was detected in APP^−/−^ mice (Fig. S2a). These results suggest a transient increase of bone resorption in the neonatal age of APP^−/−^ mice. Further examining TRAP^+^ OCs in 1-M and 2-M old mice also showed a transient increase in TRAP^+^ OCs per unit bone surface area in 1-M, but not 2-M, old APP^−/−^ mice (Fig. S2b-c). Moreover, TRAP^+^ multi-nuclei cells (MNCs), which were in vitro differentiated from BMMs of WT and APP^−/−^ mice (1-M old), were increased in APP^−/−^ cultures in response to RANKL treatment for 3–7 days, but not 7 days after RANKL treatment (Fig. S2d, e). These results suggest that APP may play an inhibitory role in OC-genesis and function transiently and/or age dependently, which may contribute in part to the osteoporotic deficit in young APP^−/−^ mice.

### Reduction of bone formation in APP^−/−^ mice

**Fig. 2:**
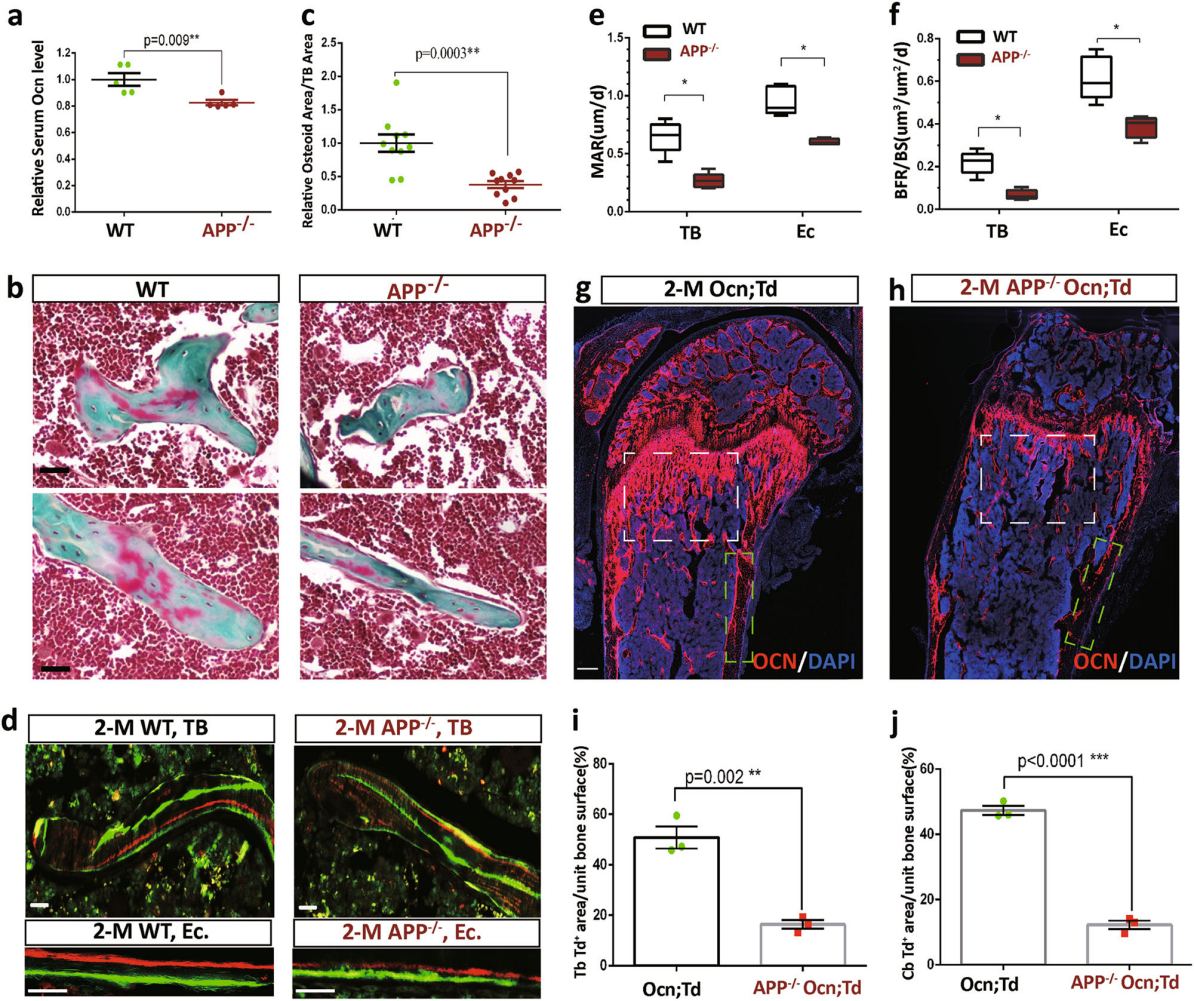
Decrease of bone formation in APP^−/−^ mice. Five different mice (2-M old, males) per genotype per assay were examined. **a** Decreased serum levels of osteocalcin, measured by ELISA assays, in APP^−/−^ mice were presented. **b**, **c** Decreased osteoid numbers of trabecular bones in APP^−/−^ femurs were viewed by Goldner’s trichrome staining analysis. Images were shown in **b**, scale bar, 20 μm, and quantification analysis of osteoid numbers per unit of bone surface was presented in **c**. **d**–**f** Reduced bone formation in APP^−/−^ mice was detected by dynamic histomorphometric measurements of double fluorescent labeled femurs. WT and APP^−/−^ mice at age of P48 were injected (intraperitoneal) with fluorochrome labeled calcein green (10 mg/kg, Sigma–Aldrich), and 10 days after (P58), they were re-injected with Alizarin red S to label active bone forming surfaces. At P60, mice were sacrificed and the left femur is fixed in 70% ETOH, sectioned at 20 μm, and viewed by fluorescence microscope (**d**), scale bar, 20 μm. The endocortical mineral apposition rate (Ec. MAR) calculated in μm/day and the bone formation rate (Ec. BFR = MAR x minerization surface/bone surface) from fluorochrome double-labels at the endocortical surfaces were illustrated in **e**, **f**, the data were shown as Min to Max with mean from five different animal per genotype. **g**–**j** Reduction in tdTomato^+^ OB-lineage cells in APP^−/−^ femurs. Femurs from Ocn-Cre; Ai9 (Ocn;Td, control) and APP^−/−^; Ocn-Cre; Ai9 mice (2-M old) were sectioned and imaged by confocal microscope. Representative images were shown in **g**, **h**, Scale bar, 300 µm; and the quantification of Td fluorescence intensity in Tb and Cb were presented in **i**, **j**. In **a**, **c**, **i**, **j**, the values of mean ± SEM from 3 to 5 different animals per genotype were shown. *p < 0.05; **p < 0.01; ***p < 0.0001

Next, we examined OB-mediated bone formation by the following assays. First, measuring serum levels of osteocalcin (a maker for bone formation) with ELISA assay showed a reduction in APP^−/−^ mice (at various ages, including 1-M, 2-M, and 5-M old) (Fig. 2a and data not shown), suggesting an age-independent decrease in bone formation. Second, Goldner’s trichrome staining analysis displayed a decrease in osteoid numbers per unit bone surface in APP^−/−^ femurs (Fig. 2b, c), in line with the view for APP to promote OB function. Third, dynamic measurements of non-decalcified femur and tibia sections, which were double-labeled by two injections of fluorescent calcein green and alizarin red S separately (at 10-day interval), showed significant reductions in MAR (mineral apposition rate) and BFR (bone formation rate) in endocortical (Ec.) bone and trabecular bone (TB) in APP^−/−^ mice (2-M old), as compared with that of same aged WT controls (Fig. 2d–f), providing additional evidence for APP’s function in bone formation. Finally, we compared OB-mediated bone formation between control and APP^−/−^ mice by use of the Ocn-Cre;Ai9 (Ocn;Td) reporter mice, in which, tdTomato’s expression depends on osteocalcin (Ocn) promoter driven Cre. In agreement, the tdTomato^+^ OBs in both trabecular and cortical bone regions were marked reduced in APP^−/−^ mice, compared with controls (Ocn-Cre;Ai9) (Fig. 2g–j). Collectively, these results demonstrate a positive role of APP in OB-mediated bone formation, which may contribute in large to the osteoporotic deficit in young as well as in aged APP^−/−^ mice.

### Decreases of OB-differentiation and function in APP^−/−^-BMSC cultures

**Fig. 3:**
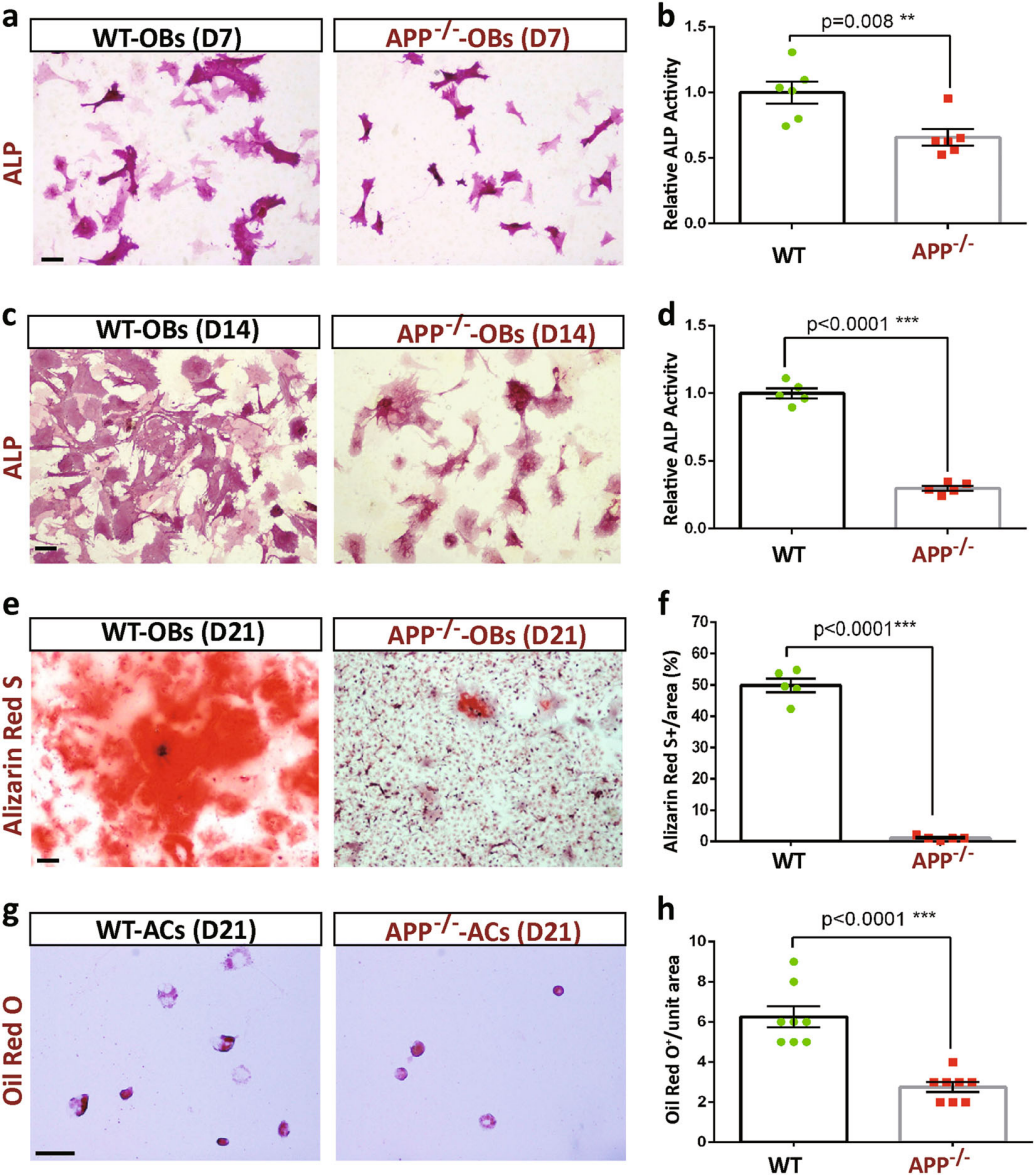
Reduced in vitro OB and adipocyte formation and function in APP^−/−^-BMSC cultures. BMSCs from 2-M old WT and APP^−/−^ femurs were induced for OB and adipocyte differentiation (see Methods). ALP staining images at day (d) 7 and 14 cultures were shown in **a**, **c**, and ALP activities (ALP positive area/over total area) were presented in **b**, **d**. At D-21 culture, OB cells were stained for Alizarin Red S (**e**), and the Alizarin Red S staining data was quantified and illustrated in **f**; and adipocytes were stained for Oil Red O (**g**), and Oil Red O staining data was quantified and presented in **h**. Scale bar, 20 μm. In **b**, **d**, **f** and **h**, the values of mean ± SEM (*n* = 5) were presented. **p < 0.01; ***p < 0.0001

To determine if APP regulates OB-mediated bone formation in a cell autonomous manner, in vitro OB differentiation assay by culturing BMSCs derived from 2-M-old WT and APP^−/−^ mice were carried out. Indeed, OB-differentiations, viewed by ALP (alkaline phosphatase) enzymatic activity staining, were lower in both D7-OB and D14-OB cultures from BMSCs of APP^−/−^ mice than those of WT controls (Fig. 3a, d). Also decreased was calcified bone matrix stained by Alizarin Red S in APP^−/−^-OB cultures (Fig. 3e–f). These results suggest a cell autonomous function of APP in promoting OB-differentiation and function.

A reduced OB-differentiation is frequently associated with an increased adipogenesis^15,19^. Examining adipocyte differentiation (viewed by Oil Red O staining) in WT and APP^−/−^ BMSC cultures, however, showed no increase, but decrease, in APP^−/−^ cultures (Fig. 3g, h). These results implicate APP’s functions in both OBs and adipocytes, rather than regulating early stage of BMSC-cell fate differentiation.

### Increases of apoptosis, ROS, and mitochondrial cytochrome C release in APP^−/−^-BM-OB cultures

**Fig. 4:**
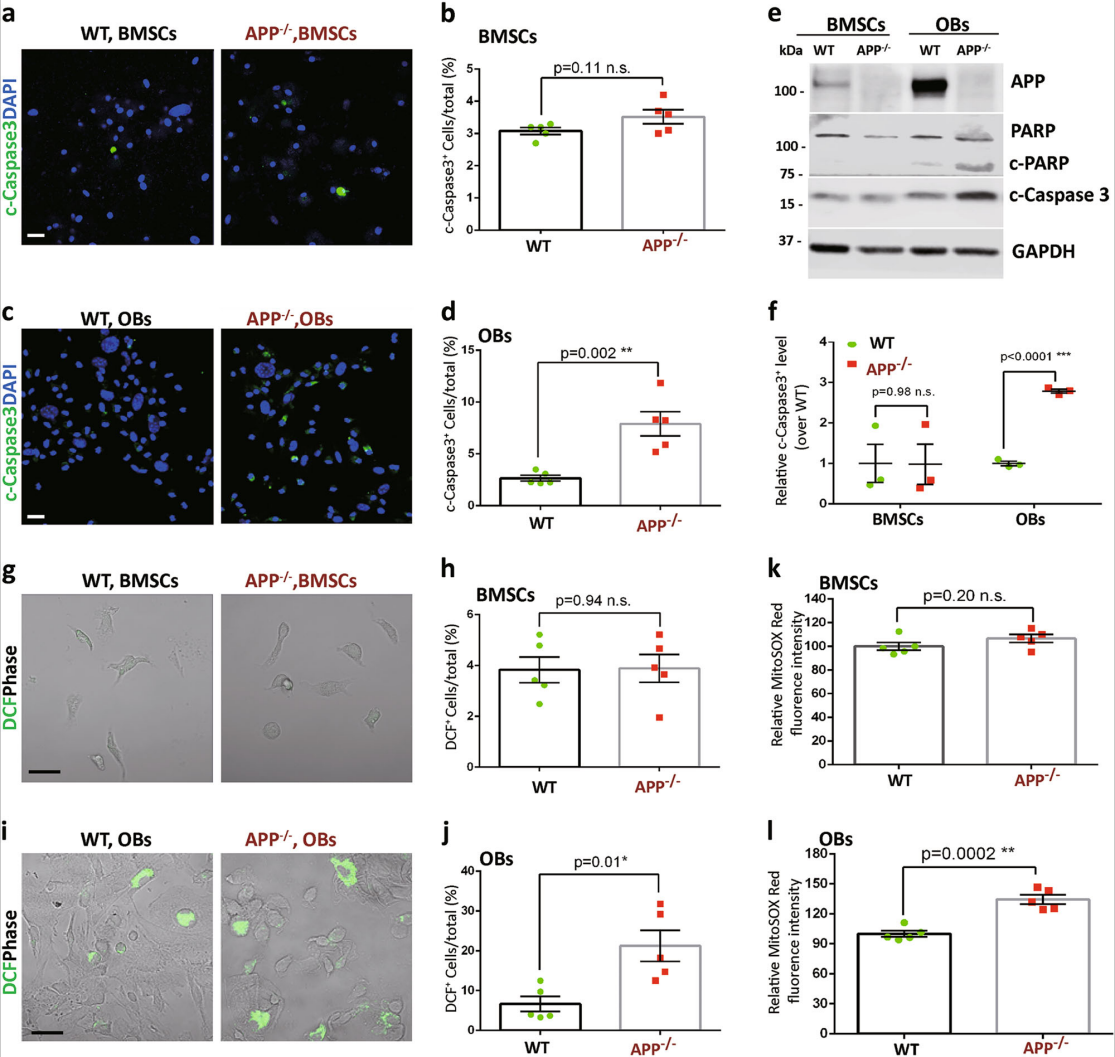
Increased apoptosis and ROS in APP^−/−^ OBs. BMSCs from 2-M old WT and APP^−/−^ femurs were induced for OB differentiation (see Material and methods section). **a**–**d** Immunostaining of cleaved (c)-caspase 3 were carried out in BMSCs (**a**, **b**) and OBs (D14 culture) (**c**, **d**). Representative images were shown in (**a**, **c**), quantification data were shown in **b**, **d**. **e**, **f** Increased c-caspase 3 and cleaved (c)-PARP1 in APP^−/−^ OBs, but not BMSCs. BMSCs and OBs from WT and APP^−/−^ mice (2-M old) were subjected to Western bot analysis using indicated antibodies. **e** Representative blots; **f** Quantifications of c-caspase 3 (compared with WT controls); Data presented were mean ± SEM (*n* = 3 mice/genotype), *p < 0.05, ***p < 0.0001. **g**–**j** DCF staining (an indicator of ROS) analyses were carried out in BMSCs (**g**, **h**) and OBs (D14 culture) (**i**, **j**). Representative images were shown in (**g, i**), quantification data were shown in **h**, **j**. Scale bar, 20 μm. The values of mean ± SEM (*n* = 5 different cultures) were presented in **b**, **d**, **h**, and **j**. **k**, **l** Increase mitochondrial ROS level in APP^−/−^ OBs. For measurement of mitochondrial O_2_^•‾^ production, WT and APP^−/−^ BMSCs and OBs were loaded with 5 μM MitoSOX (Invitrogen) in PBS containing 1 g/l glucose for 10 min at 37 °C, washed with PBS and fluorescence emission at 595 nm under 510 nm excitation was recorded using a microplate reader. Relative MitoSOX Red fluorescence intensity over WT controls were shown in **k**, **l**. **p < 0.01, ***p < 0.0001

OB migration and proliferation are important events during osteogenesis, but APP^−/−^ has no/little effect on these events (Fig. S3). Notice that a more server reduction in OB-differentiation (viewed by ALP staining) was detected in D14- than that of D7-APP^−/−^-OB cultures (Fig. 3a–d); and a more server OB functional deficit (viewed by Alizarin Red S) than that of OB-differentiation defect was also observed in APP^−/−^ cultures (Fig. 3a–f). These observations implicate APP’s function at the late stage during the OB-differentiation assay. We thus wondered if APP is critical for OB-survival. Cell death assay by immunostaining analysis with antibodies against active caspase 3, a marker for apoptosis, was carried out. Indeed, cleaved (c)-caspase 3^+^ cells were much more in APP^−/−^ OB cultures than that of controls by both immunostaining and Western blot analyses (Fig. 4a–f), indicating an increase of OB apoptosis. Notice the c-caspase 3 was not increased in APP^−/−^ BMSCs (Fig. 4a–f), demonstrating a selective effect of APP. This view was further supported by the observations that the cleaved (c)-PARP1 (another marker of apoptosis) and ROS (reactive oxidative species), a cell death inducer viewed by both DCFDA fluorescence and MitoSOX Red, were all increased in APP^−/−^ OBs, but not BMSCs (Fig. 4e–l).

**Fig. 5:**
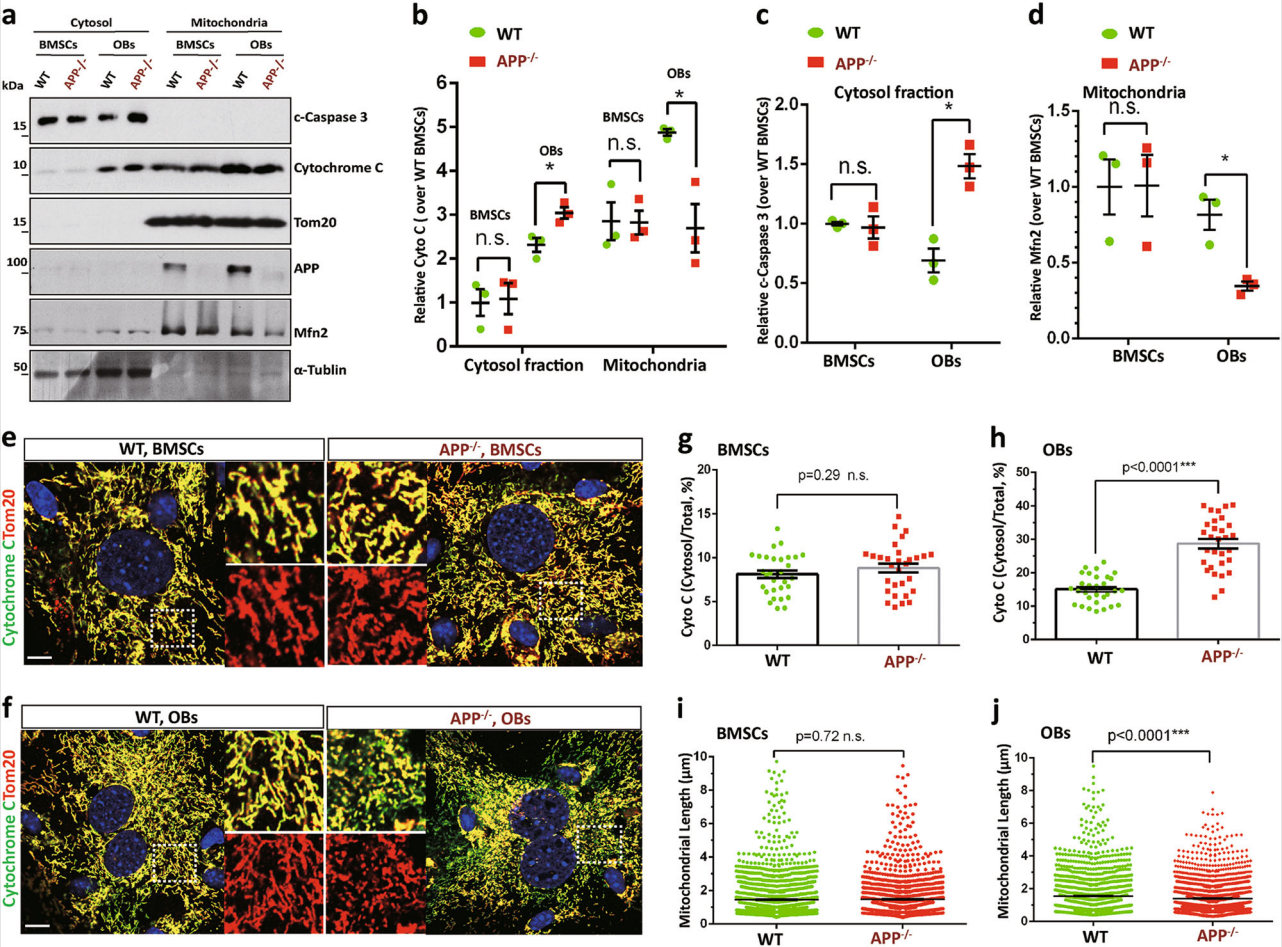
Increased cytochrome C release and mitochondrial fragmentation in APP^−/−^ OBs, but not BMSCs. **a**–**d** Reduced mitochondrial cytochrome C and Mfn2 in APP^−/−^ OBs, but not BMSCs. Mitochondrial and cytosol fractions of BMSCs and OBs from WT and APP^−/−^ mice (2-M old) were subjected to Western bot analysis. a, Representative blots; **b**–**d** Quantifications of protein levels (compared with WT BMSCs); Data presented were mean ± SEM (*n* = 3 mice/genotype); *p < 0.05. **e**–**j** Increased cytosolic cytochrome C and mitochondrial fragmentation in APP^−/−^ OBs, but not BMSCs. OBs (D14 culture) were in vitro differentiated from BMSCs from WT and APP^−/−^ mice (2-M old). Both BMSCs and OBs were co-immunostained with indicated antibodies. Mitochondria were labeled by Tom20. Representative images were shown in **e**, **f**. Higher power views of mitochondria (Tom20) and cytochrome C were also included in **e**, **f** Scale bar, 5 μm. **g**, **h** Quantifications of cytochrome C fluorescence in cytosol over total. The cytosolic cytochrome C was defined by cytochrome C^+^ Tom20^−^ staining. Data presented were mean ± SEM (*n* = 30 cells from 3-different cultures). **i**, **j** Quantifications of mitochondrial length. Shown were grouped column scatter (with mean ± SEM). *n* = 1500–2000 mitochondria from 30 different cells of each group. ***p < 0.0001

To understand how APP regulates OB apoptosis, we examined the canonical apoptotic pathway-activation of caspase 3 by mitochondrial released cytochrome C^20^. Mitochondrial and cytosol fractions were purified from lysates of BMSCs and OBs. APP was largely distributed in mitochondria^21^(Fig. 5a and Fig. S4). Western blot analysis showed an increase of cytochrome C in cytosol fractions of OBs, but not BMSCs, from APP^−/−^ mice, as compared with those of WT controls (Fig. 5a–b). The cleaved caspase 3 was also elevated in the cytosol fractions of APP^−/−^ OBs (Fig. 5a, c). The increased cytosolic cytochrome C in APP^−/−^ OBs, but not BMSCs, was further examined by co-immunostaining analysis using antibodies against cytochrome C and Tom20 (a mitochondrial outer membrane protein). As shown in Fig. 5e–h, the mitochondrial cytochrome C was lower, but the mitochondrial un-associated cytochrome C was higher, in APP^−/−^ OBs, compared with that of WT controls. Together, these results demonstrate an increase of cytochrome C release from mitochondria of APP^−/−^ OBs, and revealing an underlying mechanism of APP preventing OBs from cell death.

In addition to the increased mitochondrial cytochrome C release, the mitochondrial morphology (labeled by Tom20) appeared to be shorter and rounder in APP^−/−^ OBs than those in control OBs but not BMSCs (Fig. 5e–j), suggesting mitochondrial fragmentation in APP^−/−^ OBs. This view was further supported by the observation of a decrease of mitofusin 2 (Mfn2), a GTPase critical for mitochondrial fusion, in mitochondrial fractions of APP^−/−^ OBs (Fig. 5a, d). These results suggest that APP in OBs may be required for mitochondrial fusion dynamics.

### Mitochondrial defects in APP^−/−^-BM-OB cultures

**Fig. 6:**
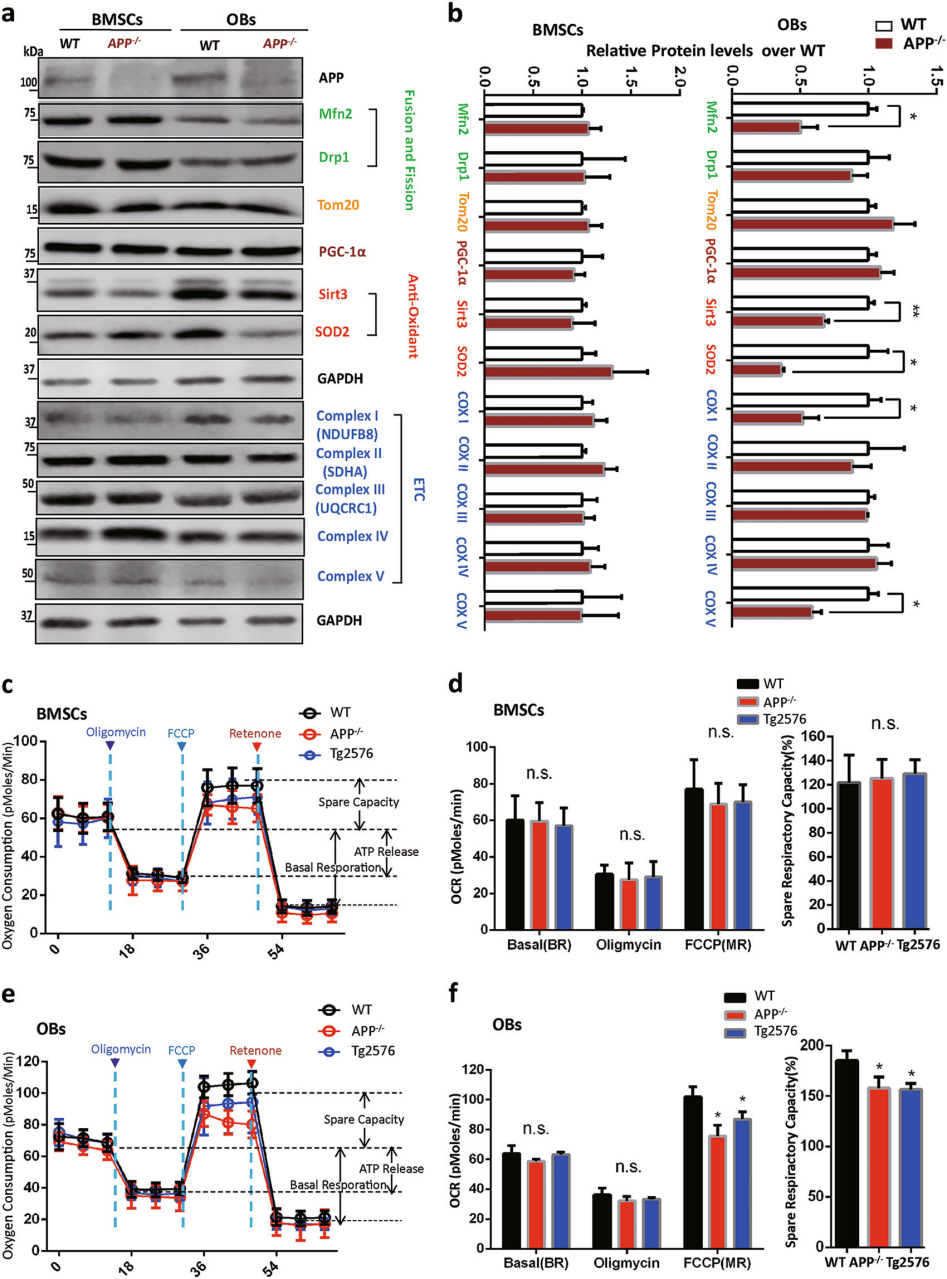
Mitochondrial defects in APP^−/−^-BM-OB cultures. **a**, **b** Western blot analysis of mitochondrial proteins using indicated antibodies. **a** Representative blots; **b** Quantifications of protein changes in APP^−/−^ mice (compared with WT); Data presented were mean ± SEM (*n* = 3 mice/genotype); *p < 0.05, **p < 0.01. **c**–**f** Mitochondrial functional deficits in APP^−/−^ and Tg2576-OBs, but not their BMSCs, by a Seahorse XF96 analyzer. OBs (D14 culture) were in vitro differentiated from BMSCs of 2-M old WT, APP^−/−,^ or Tg2576 femurs. Both BMSCs (**c**, **d**) and OBs (**e**, **f**) were subjected to the measurements of the real-time O_2_ consumption rate (OCR) by the Seahorse XF96 analyzer. In **c**–**f**, to validate the OCR measurement, ATP synthase inhibitor, oligomycin (2 µM), was treated after recording the basal line, and the pharmaceutical uncoupler, FCCP (3 µM), and the Complex I inhibitor rotenone (1 µM) were then treated as indicated. Representative traces of OCR were shown in **c**, **e**. Quantifications were presented in **d**, **f**. The spare respiratory capacity changes (compared with BR) were also showed in the right columns of d, f. The values of mean ± SEM (*n* = 3) were shown. *p < 0.05

Mitochondria are the major source of ROS and ATP production, which are increased during OB genesis^22^. To understand how APP prevents ROS, we examined the following mitochondrial proteins, in addition to Mfn2, in WT and APP^−/−^ BMSCs and OBs. Sirt3 (a mitochondrial deacetylase Sirtuin 3) and Sod2 (superoxide dismutase 2) are both critical proteins for anti-oxidant response^23,24^ and OB-mediated bone formation^22^_._ Drp1 (dynamin-related protein 1) is a key protein for mitochondrial fission^25–28^. Mitochondrial complex proteins (I, II, III, IV, and V) are essential for ATP production from ETC (electoral transfer chain) reaction. Remarkably, Sirt3, Sod2, Mfn2, but not Drp1 or Tom20, were reduced in APP^−/−^ OBs, but not BMSCs (Fig. 6a, b). In addition, the complex I (subunit UQCRC1) and V, but not II (subunit SDHA), III and IV, were also lower in APP^−/−^ OBs (Fig. 6a, b). These results suggest a reduction in Sirt3/Sod2 mediated anti-oxidant response and impairments in mitochondrial fusion and ETC function in APP^−/−^ OBs, which are likely to contribute to the increase of ROS.

Considering the importance for PGC-1alpha for mitochondria biogenesis and function, PGC1alpha was also examined. It was comparable in APP^−/−^ OBs or BMSCs to that in WT OBs/BMSCs (Fig. 6a, b), suggesting little role, if there is any, that APP regulates PGC1α expression.

We next examined mitochondrial function (respiratory capacity) in APP^−/−^ BMSCs and OBs by the Seahorse plate form assay to real time measure oxygen (O_2_) consumption rates (OCR). No difference in rates of the basal oxygen consumption (BR) or oxygen consumption (OCR) was detected between WT and APP^−/−^ OBs (Fig. 6e, f). But, the maximal respiratory rate (MR) induced by mitochondrial un-coupler (Carbonyl cyanide-p-trifluoromethoxyphenylhydrazone (FCCP)) treatment was reduced in APP^−/−^ OBs (Fig. 6e, f), resulting in a deficiency in spare respiratory capacity (SR) (Fig. 6f). Again, this deficit was detected only in APP^−/−^ OBs, but not in APP^−/−^ BMSCs (Fig. 6c, d), demonstrating dysfunctional mitochondria in cultured APP^−/−^ OBs.

As similar osteoporotic deficits were observed in Tg2576 mice^14,15^, we asked if OBs from Tg2576 mice show any mitochondrial deficit as that of APP^−/−^ OBs. Indeed, the SR was lower in OBs from Tg2576 mice (Fig. 6e, f), supporting the view for APPswe acting as a dominant negative regulator in this event.

In addition to mitochondrial function in ATP production, we examined glycolysis, an oxygen independent pathway for ATP production, in WT and APP^−/−^ BMSCs and OBs (Fig. S5). The ATP level by the glycolytic pathway, but not mitochondrial oxidative phosphorylation (OXPHO)-pathway, was reduced in APP^−/−^ BMSCs (Fig. S5a–b), suggesting a deficit in glycolysis. However, in APP^−/−^ OBs, whereas OXPHOS-dependent ATP production was reduced (Fig. S5c), the glycolysis regulated ATP level was increased (Fig. S5d), in line with the reports that blocking OXPHOS pathway results in a compensatory increase of ATP production by the glycolytic activation^29,30^. The reduction of OXPHOS-dependent ATP production in APP^−/−^ OBs was in line with the results from the Seahorse assay. Together, these results demonstrate an impaired oxygen or mitochondrial dependent ATP production in APP^−/−^ OBs, but not BMSCs.

### Increased mitophage/autophage in APP^−/−^ OBs and APP-KO MC3T3 cells

**Fig. 7:**
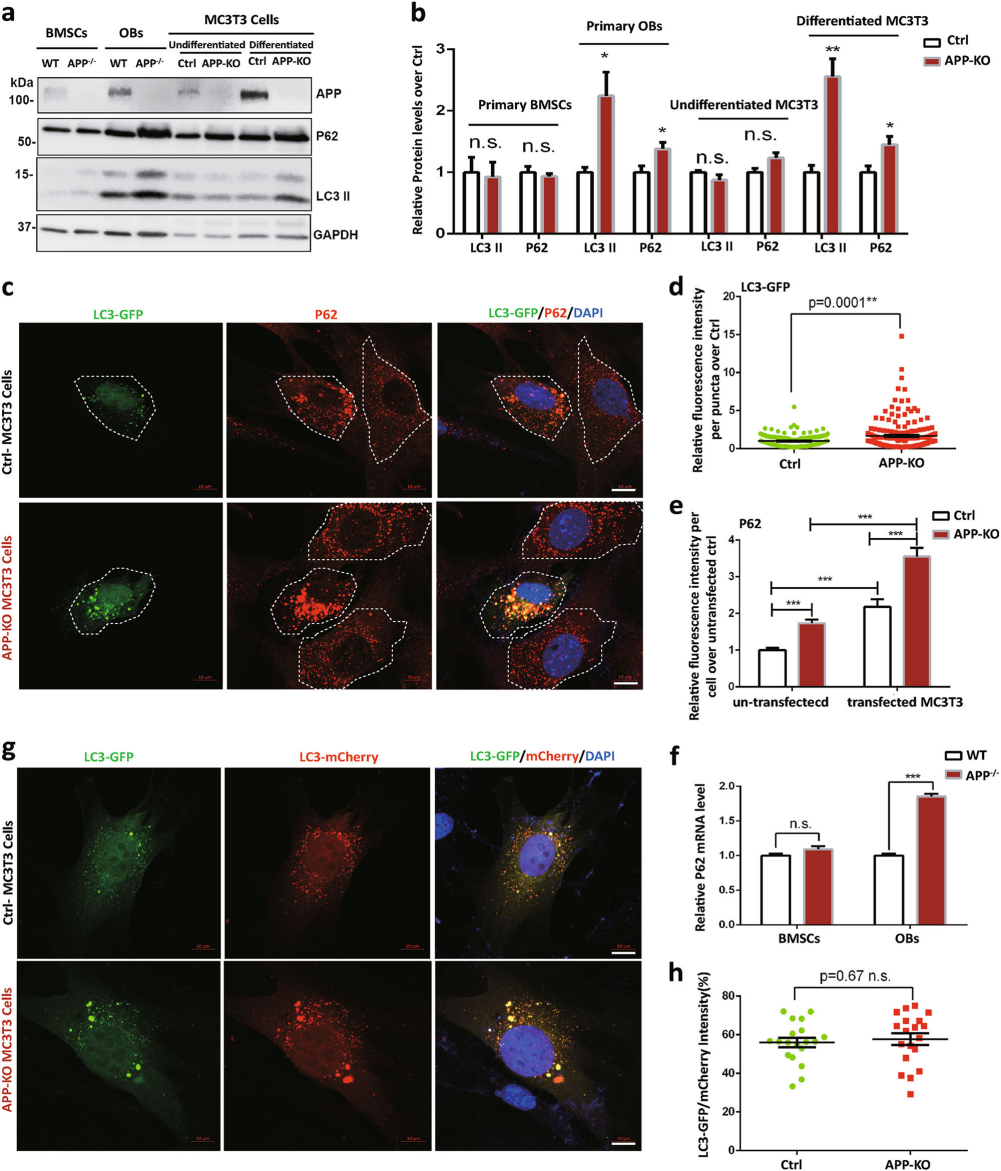
Increased LC3 II and P62 proteins in APP^−/−^ OBs and differentiated APP-KO MC3T3 cells. **a**, **b** Western blot analysis of proteins using indicated antibodies. **a** Representative blots; **b** Quantifications of relative protein levels. Data presented were mean ± SEM (*n* = 3); *p < 0.05. **c**–**e** Increased LC3 and P62 fluorescence intensities in differentiated APP-KO MC3T3 cells. To differentiate pre-osteobalstic MC3T3 cells to mature osteoblastic MC3T3 cells, both control and APP-KO MC3T3 cells were induced by the addition of 50 ng/ml ascorbic acid and 10 mM β-glycerophosphate to standard growth medium for 7 days. Then those differentiated cells were transfected with GFP-LC3 plasmid. Seventy-two hours after transfection, cells were fixed and immunstained with indicated antibodies. **c** Representative images. Scale bar, 10 µm; **d**, **e** quantification analyses of fluorescence intensity of LC3-GFP puncta (**d**) and endogenous P62 per cell in un-transfected and transfected MC3T3 cells (**e**). Data presented were mean ± SEM, *n* = 20 from three-different cultures, **p < 0.01, ***p < 0.0001. **f** RT-PCR analysis of P62 mRNA levels in WT and APP^−/−^ BMSCs and OBs. Data presented were mean ± SEM (*n* = 3); ***p < 0.0001. **g**, **h** Normal autophagosome-lysosome fusion in APP-KO MC3T3 cells. Differentiated control and APP-KO MC3T3 Cells were transfected with LC3-mCherry-GFP fusion plasmid. Three days after transfection, Cells were then subjected to fluorescence imaging analysis. **g** Representative images. Scale bar, 10 µm; **h** Quantification analysis. Data presented were ratios of GFP/mCherry intensities, mean ± SEM (*n* = 18). *p < 0.05

In addition to mitochondrial fission, reduced Mfn2 is frequently associated with increased mitophage, a critical pathway for mitochondrial degradation/removal by autophage^31^. We thus examined LC3-II and P62/SQSTM1, both autophagic proteins, in APP^−/−^ BMSCs and OBs (Fig. 7a, b). Indeed, both LC3-II and P62 were elevated in APP^−/−^ OBs, but not BMSCs (Fig. 7a, b), implicating an increase in mitophage.

To further test this view, we generated APP-KO MC3T3 cells (an OB cell line) by Crispr-Cas9 method (Fig. 7a) and examined LC3-II and P62 levels in these cells by both Western blot and immunostaining analyses. Remarkably, both LC3-II and P62 were higher in differentiated APP-KO MC3T3 cells than those of control cells (Fig. 7a, b). Immunostaining analysis of MC3T3 cells expressing exogenous GFP-LC3 showed enlarged LC3^+^ vesicles in APP-KO MC3T3 cells (Fig. 7c, d). Interestingly, the endogenous P62 was increased not only in APP-KO cells (compared with controls), but also in GFP-LC3 expressing cells (compared with un-transfected cells) (Fig. 7c, e). P62 mRNA levels were also higher in APP^−/−^ OBs than that of WT controls (Fig. 7f). Furthermore, MC3T3 cells expressing exogenous GFP-LC3 and Mito-RFP displayed more co-localization of GFP-LC3 with Mito-RFP in APP-KO cells than that of control cells (Fig. S6a–c). However, MC3T3 cells expressing LC3-eGFP-mCherry fusion protein showed comparable ratio of GFP/mCherry between control and APP-KO cells (Fig. 7g, h), suggesting little role of APP in autophagic-lysosome fusion or autophagic flux. Together, these results support the view for an increased mitophage/autophage formation in APP^−/−^ OBs.

Mfn2 is also believed to be an element for the maintenance of a correct mitochondria-associated-ER membrane (ER-MAM) juxstapostition^32,33^. We measured mitochondrial-ER contact sites by imaging and quantification analysis of the co-localization of Mito-RFP (a mitochondrial marker) with Sec61b-GFP (an ER marker). No significant difference was detected between WT and APP^−/−^ OBs (Fig. S6d–f).

### Diminished deficits in mitochondrial function, OB-differentiation, and bone homeostasis in APP^−/−^ OBs and mice treated with NAC

**Fig. 8:**
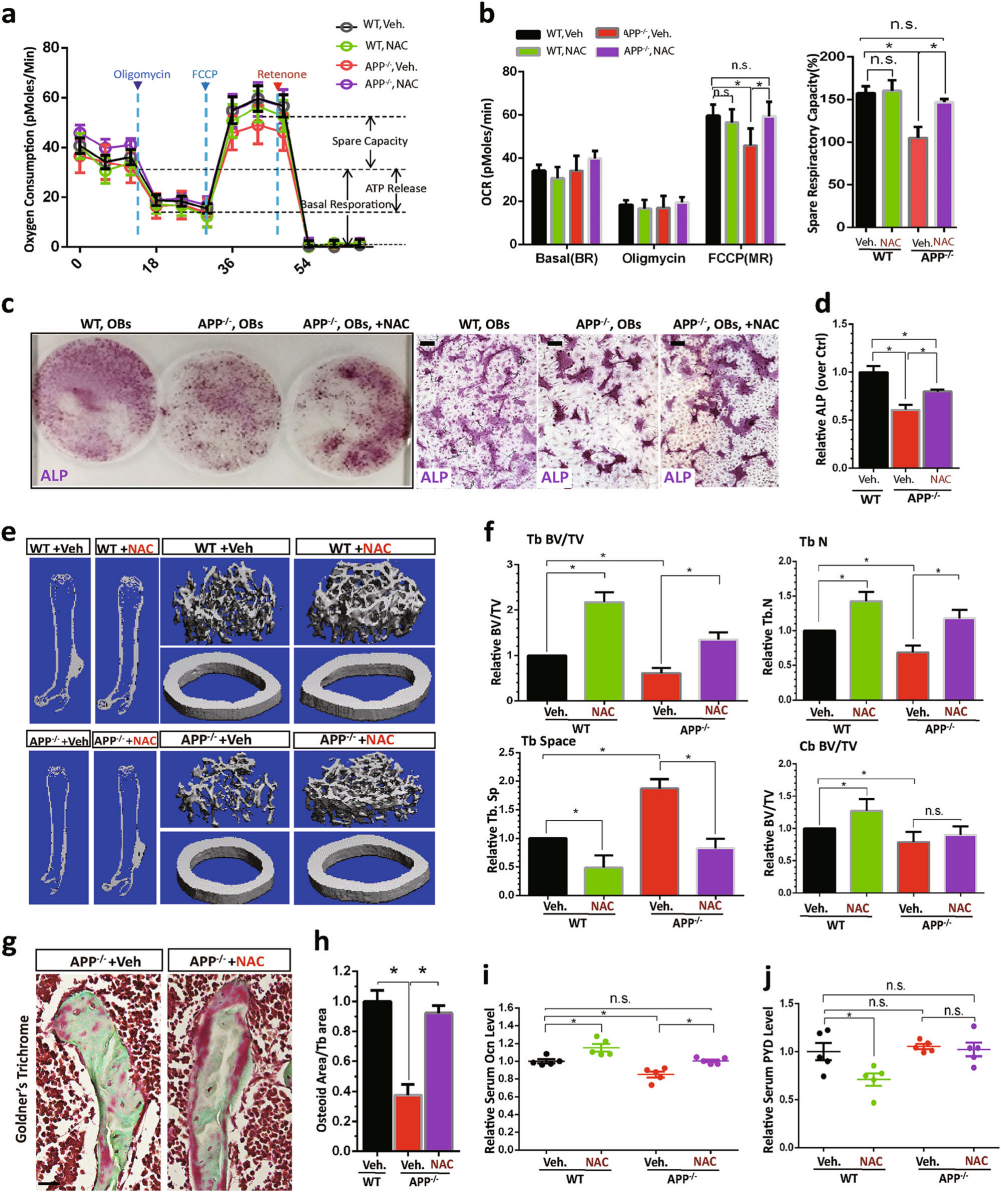
Diminished deficits in mitochondrial function, OB-differentiation, and bone homeostasis in APP^−/−^ OBs and mice treated with NAC. **a**, **b** NAC restore of mitochondrial function of APP^−/−^ OBs. OBs (D1 culture) were treated with NAC (1 mM, overnight) or vehicle (veh) control. At D14, OBs’ mitochondrial function was evaluated by a Seahorse XF96 analyzer. Representative traces of OCR were shown in **a** Quantifications were presented in **b**. The values of mean ± SEM (*n* = 3) were shown. *p < 0.05. **c**, **d** NAC restore of OB differentiation (viewed by ALP staining) from BMSCs of APP^−/−^ mice. As **a**, **b**, D1-OB cultures were treated with NAC (1 mM) or veh control, and D14-OBs were stained with ALP. Representative images were shown in **c**, scale bar 20 μm, and the quantification analysis of the average ALP activities (normalized by WT control) was presented in **d**. The values of mean ± SEM from three different cultures were presented. *p < 0.05. **e**–**j** APP^−/−^ mice (at age of 1-M old, 5 per group, male) were fed with drinking water containing with vehicle (Veh.) or NAC (2 mg/kg/day) for 3 months, and their femur bone samples and sera were collected for phenotypic analyses. **e**, **f** The μCT analysis displayed an increased trabecular bone volumes of NAC-treated APP^−/−^ femurs. The trabecular bone thickness (Tb.Th) and trabecular separation (Tb.Sp) were all ameliorated or improved by NAC treatment. The cortical bone volumes were unaffected by NAC. Scale bar, 1 mm. **g**, **h** Goldner’s trichrome staining analysis showed an increased osteoid numbers of trabecular bones in NAC-treated APP^−/−^ femurs. Representative images were shown in **g**, scale bar, 20 μm, and the data were quantified (osteoid area/bone area, normalized by WT) and showed in **h**. **i** Increased serum levels of osteocalcin in NAC-treated APP^−/−^ mice were detected by ELISA assays. **j** No change of serum levels of PYD in NAC-treated APP^−/−^ mice. In **f**, **h**–**j**, the values of mean ± SEM from five different animals per genotype were shown. *p < 0.05

Increased ROS is believed to be detrimental for AD pathogenesis^34^. Both AD-like and skeletal ageing-like phenotypes in Tg2576 mice can be ameliorated by treatments with anti-oxidant(s), NAC, a glutathione (GSH) precursor that increases GSH levels and reduces free radicals^15,35^. The similar osteoporotic and mitochondrial deficits between APP^−/−^ and Tg2576 mice lead us to ask if NAC could also diminish the bone deficits in APP^−/−^ mice. To this end, we first tested NAC’s effect on mitochondrial and OB-differentiation deficits in cultured APP^−/−^ OBs. As expected, the impaired spare capacity of mitochondria of APP^−/−^ OBs was diminished when NAC was present in the OB cultures (Fig. 8a, b). NAC increased ALP positive OBs from APP^−/−^ BMSC cultures, partially restoring the OB-differentiation (Fig. 8c, d).

We next tested NAC’s effect in vivo. APP^−/−^ mice (at age of 1–2 M old) were fed with drinking water containing vehicle or NAC (2 mg/kg/day) for 3-M and their femur bone and sera samples were then collected for phenotypic analysis. As shown in Fig. 8e, f, bone volume, particular trabecular bone volume, was increased in NAC-treated APP^−/−^ femurs, compared with that of vehicle treatment, based on μCT analysis. Bone formations, viewed by Goldner’s trichrome staining of osteoid density and by measurement of serum levels of osteocalcin, were also elevated in NAC-treated APP^−/−^ mice (Fig. 8g–i). These results thus demonstrate an efficient amelioration of the osteoporotic deficit by NAC. In addition to NAC’s effect on bone formation, we examined its role in bone resorption in WT and APP^−/−^ mice. Bone resorption, indicated by serum levels of PYD, was reduced by NAC treatment of WT, but not APP^−/−^, mice (Fig. 8j). Taken together, these results suggest that NAC’s amelioration of the osteoporotic deficit in APP^−/−^ mice may be due in large to its anti-oxidant effects on OBs.

## Discussion

This study provides evidence for APP to be necessary for OB survival and bone formation, and thus maintaining bone homeostasis. Mechanistically, the osteoblastic APP appears to be critical for mitochondrial function, and thus preventing from cytochrome C release, ROS production, and apoptosis.

APP regulation of bone homeostasis is evident based on μCT and histological analyses of long bone structures of APP^−/−^ mice. This function appears to be largely due to APP-promotion of OB-mediated bone formation. Interestingly, there is a remarkable similarity in bone deficits between APP^−/−^ and Tg2576 mice. Both show decreases in OB-genesis, bone formation, and bone mass, and transiently increases in OC-genesis and bone resorption in young age. These observations implicate that APPswe may act as a dominant negative regulator of endogenous APP in both OB and OC cells.

How does APP regulate OB-function? We posit that APP enhances OB-function likely by promoting OB mitochondrial function, preventing ROS production and cytochrome C release, and thus increasing OB survival, in light of the following observations. First, the early stage of OB differentiation from BMSCs and OB progenitor proliferation appeared to be normal in APP^−/−^ cells (Fig. S3c-d); however, the late stage of OB differentiation and function, which depend on OB-survival, were severally impaired in APP^−/−^ OB cultures (Fig. 3). Second, OB cell death markers, including cleaved caspase 3, cleaved PARP1, cytochrome C release, and ROS, were all elevated in APP^−/−^ OBs, but not BMSCs (Figs. 4, 5). Third, mitochondrial morphological and functional deficits in APP^−/−^ OBs, but not BMSCs, were evident. Critical proteins for anti-oxidant response (e.g., SIRT3 and SOD2) and proteins for mitochondrial ETC functions (e.g., complex I, and V) were all reduced in APP^−/−^ OBs (Fig. 6a, b); mitochondrial SR (viewed by Seahorse plate form assay) and OXPHOS-dependent ATP production were much lower in APP^−/−^ OBs (Fig. 6e, f, Fig. S5); and mitochondrial fusion (viewed by immunostaining analysis of its morphology and western blot analysis of MFN2) was also impaired in APP^−/−^ OBs (Figs. 5 and 6a, b). Finally, treatments with anti-oxidant NAC in APP^−/−^ BMSC-OB cultures restore mitochondrial function and OB-differentiation (Fig. 8a–d), and NAC treatment in APP^−/−^ mice increased bone formation and diminished the osteoporotic deficit (Fig. 8c–j).

APP regulation of mitochondrial function is also supported by the following observations. It is largely distributed in mitochondria^21^ (Fig. 5a and Fig. S4); Aβ disrupts mitochondrial fusion and function^36^; and over expression of APPswe in various cell lines results in mitochondrial fusion deficits (data not shown). Why mitochondrial function is so important for OB-mediated bone formation? Notice that osteogenic induction is accompanied by robust oxygen consumption; and mitochondrial biogenesis is increased during OB-differentiation^22^. In line with this view are our observations of much higher levels of mitochondrial anti-oxidant (Sirt3 and Sod2) and Cytochrome C proteins in OBs than those in BMSCs (Figs. 5a, b and 6a); and APP was also higher in OBs than that in BMSCs (Figs. 5a and 6a).

The increased autophagic proteins, LC3-II and P62, in APP^−/−^ OBs (Fig. 7a, b) suggest a role that APP may play in autophagosome formation. Considering no obvious deficits in mitochondrial protein degradation (Fig. 6a, b) and autophage-lysosome fusion (Fig. 7g, h) in APP^−/−^ OBs, the increased P62 is thus likely due to LC3-mediated autophagosome formation and ROS-induced P62 expression^37,38^. This view is also supported by the observations that expressing GFP-LC3 in MC3T3 cells increased endogenous P62 (Fig. 7c–e) and P62 transcripts are increased in APP^−/−^ OBs (Fig. 7f).

How does APP regulate OB mitochondrial function? APP in mitochondrial membrane may play a role in maintaining the stability of Mfn2 and Sirt3/Sod2, promoting Mfn2-mediated mitochondrial fusion and function and Sirt3/Sod2 mediated anti-oxidant response, and thus preventing ROS production, an inducer of OB apoptosis and an inhibitor of OB differentiation and function. Additionally, APP in mitochondrial membrane may prevent Cytochrome C release from mitochondria, and thus inhibiting caspase 3-driven OB apoptosis. This view is in agreement with the observations that treatment with NAC ameliorates the osteoporotic deficit not only in young adult Tg2576 mice, but also in APP^−/−^ mice. Interestingly, NAC pretreatment in AD animal models, including Tg2576 mice, reduces Aβ levels, decreases lipid peroxidation, and improves cognition^39^. In light of these results, we speculate that NAC may be an effective therapy in amelioration of AD-associated bone deficit.

## Materials and methods

### Reagents and animals

Antibodies including APP (#2452, rabbit polyclonal), Cleaved Caspase-3(#9664), Mitofusin-2(Mfn2) (#9482), cytochrome C (#4272 Rabbit polyclonal), PARP1(#9452) and SirT3 (Rabbit mAb #5490) were purchased from Cell Signaling Technology (Danvers, MA, USA), Ki67 (AB9260) was purchase from EMD Millipore, DRP1(NB110-55288) was purchased from Novus Biologicals, Cytochrome C (556432, mouse monoclonal) was purchase from BD Bioscience,Tom20(PA5-52843, Rabbit polyclonal), Complex I(NDUFB8, #459210), Complex II(SDHA, #459200), Complex III (UQCRC1, #459140), Complex IV(A21348) and Complex V(ATP synthase beta, A21351) monoclonal antibodies were purchased from ThermoFisher, PGC-1α(ab54481), SOD2(ab13533), P62(ab56416) and LC3 A/B (ab128025) were purchased from Abcam, Tom20 (sc-17764, mouse monoclonal), GAPDH, β-actin and α-tublin were purchased from Santa Cruz. H2DCFDA (D399) and MitoSOX Red (M36008) was purchased from Thermofisher Scientific (Carlsbad, CA, USA), N-acetyle-L-cysteine (NAC) were purchased from Sigma (St. Louis, MO, USA).

APP^−/−^ mice (stock number 004133, C57BL/6 background) were purchased from the Jackson’s laboratory (Bar Harbor, ME, USA, USA). Same aged wild type (WT) mice (C57BL/6 background) were used as controls. Male and female mice were characterized. Ocn-Cre; Ai9 (Ocn;Td) and APP^−/−^; Ocn; Ai9 mice were generated by crossing Ai9 mice (from the Jackson Laboratory, donated by Dr. Hongkui Zeng, Allen Institute for Brain Science) with Ocn-Cre transgenic mice (provided by Dr. T. Clemens, Johns Hopkins Medical School) and APP^−/−^ mice. Ai9 mice have a loxP-flanked STOP cassette preventing translation of a CAG promoter-driven red fluorescent protein variant (tdTomato). All mice were housed in a room with a 12 h light/dark cycle with ad libitum access to water and rodent chow diet (Diet 7097, Harlan Teklad). All experimental procedures were approved by the IACUC (Institutional Animal Care and Use Committee) at the Case Western Reserve University and Augusta University, in accordance US National Institutes of Health guidelines.

### In vitro BMSC, OB, and adipocyte cultures

The primary cultures of BMSCs, OBs, and adipocytes were carried out as described previously^15,40^. In brief, the whole bone marrow cells flushed out from long bones of 2–3 month old WT, APP^−/−^ mice with DMEM were filtered through a 70-mm filter mesh, washed, re-suspended, and then plated in 100-mm dishes with growth medium (DMEM plus 10% FBS), which were incubated at 37 ^o^C with 5% CO_2_. The non-adherent cells were removed 72 h after medium changing. The attached BM cells were cultured with the growth medium for 7 days. These cells were then resuspended and plated at a density of 12-well plates or culture dishes, and cultured for another 3–6 days with the same growth medium. These cells, so called BMSCs, were used for Western blot, immune-staining analysis, or subjected to further differentiation with OB or adipocyte differentiation medium. For OBs and adipocytes, BMSCs were plated in densities of 1 × 10^4^/cm^2^ and 3 × 10^4^/cm^2^ at 12-well plates for OB and adipocyte inductions, respectively. To induce OB genesis, BMSCs were exposed in osteogenic medium (DMEM containing 10% FBS, 1%P/S, 10 mM β-glycerophosphate and 50 μM l-ascorbic acid-2- phosphate). After 7–14 days, ALP (alkyline phosphatase) staining and quantification were performed as described previously^15,40,41^. At D14, the OBs were used Western blot, immune-staining analysis. After 21 days, to visualize calcified matrix, Alizarin Red S staining were performed and quantification as previous described^40^.

To induce adipogenesis, cells were maintained to adipocyte differentiation media (DMEM containing 10% FBS, 1% P/S, 0.5 mM isobutylmethylxanthine, 10 μg/ml insulin and 1 × 10^−6^ M dexamethasone) for 3 days, followed by a 9-day incubation in maintenance media (growth medium plus 10 μg/ml insulin) with media replaced every other day. The cells were monitored daily using a microscope for the appearance of lipid droplets, which were confirmed by Oil-Red O staining as described previously^15,40^. Oil Red O solution was freshly made by diluting a stock solution (0.5 g of Oil Red O in 100 ml of isopropanol) with water (6:4) followed by filtration.

### In vitro BMM and OC cultures

Mouse BMMs and OCs were generated as described previously^7,14,15,41^. In brief, the bone marrow was flushed from femurs and tibiae of 2–3 month old WT and APP^−/−^ mice with ice-cold α-MEM and plated on 100 mm tissue culture plates in α-MEM containing 10% FBS and 10 ng/ml recombinant M-CSF (macrophage colony-stimulating factor). Then cells were incubated at 37 °C with 5% CO_2_ overnight. Non-adherent cells were harvested and subjected to Ficoll-Hypaque gradient centrifugation for purification of BMMs. For osteoclastogenesis, 1 × 10^5^ BMMs were incubated with OC differentiation medium containing 10 ng/ml recombinant M-CSF and 100 ng/ml recombinant RANKL (receptor activator of NFκB ligand). Mature OC (multi-nucleated, large spread cells) began to form at day 3–4 after RANKL treatment. The cells were then subjected to TRAP (tartrate-resistant acid phosphatase) staining to confirm OC identity.

### Transwell migration assay

The migration ability of OBs isolated from long bone of WT and APP^−/−^ mice was examined in 6.5 mm transwell chambers with 8 μm pores (Sigma). Briefly, 600 μl DMEM supplemented with 10% FBS was added to the bottom chamber. Two hundred microliter resuspended cells (1 × 10^5^/ml) treated with compound in serum-free DMEM were added to the top chamber. After 8 h, insert membranes were fixed with paraformaldehyde 4% (w/v), non-migrated cells removed with cotton swabs. Then the chamber was stained with crystal violet (Sigma) for 5 min, membranes cut from the inserts and mounted using fluoroshield without DAPI. Migrated cells were counted in the entire insert membranes using a microscope (Leica Microsystems).

### Measurement of serum levels of osteocalcin and PYD

Mouse blood was obtained by cardiac puncture. Samples were allowed to clot for at least 30 min and then centrifuged for 10 min at 3000 rpm. Serum was collected and frozen at −20 °C until use. Mouse serum levels of osteocalcin were measured by using mouse osteocalcin Elisa kit (QUIDEL Corporation). The serum levels of PYD were determined by using METRA Serum PYD EIA kit (QUIDEL Corporation). All the assays were carried out the instructions. All the ODs measured after reactions were converted to osteocalcin/PYD concentration using their standard curves. All the samples were measured in duplicate, and values were subjected to statistical analysis.

### Micro-computed tomography (μCT)

Micro-architecture of the distal trabecular bone and midshaft cortical bone of the femur were measured by Scanco μCT 40 (Scanco Medical AG, Brüttisellen, Switzerland) as described previously^14,15,40–42^. The 3-D reconstruction of the trabecular bone was performed using all the outlined slices. No cortical bone was included in this analysis. Data was obtained on bone volume (BV), total volume (TV), BV/TV, bone density, trabecular number, and connectivity. The scan of the cortical bone was performed at the midshaft of the femur and consisted of 25 slices (each slice was 12 µm in thickness). Scans were reconstructed as for the trabecular scans and the region of interest was drawn on all 25 slices. There was no trabecular bone in these images at the midshaft. Cortical bone was thresholded at 329, and the 3-D reconstruction was performed on all 25 slices. Data was obtained on BV, TV, BV/TV, bone density, and cortical thickness.

### Bone histomorphometric analysis

Bone histomorphometric analyses were carried out as previously described^14,15,40–42^. In brief, mouse tibia and femurs were fixed overnight in 10% buffered formalin, decalcified in 14% EDTA for 2 weeks, embedded in paraffin, sectioned, and subjected for H&E, TRAP and Goldner’s Trichrome stain analyses.

### Bone dynamic histomorphometric analysis

To obtain the bone formation rate, P16 mice were injected with the fluorochrome labels calcein green (10 mg/kg, Sigma-Aldrich) intraperitoneally, and followed by another injection 12 days later. Mice were sacrificed two days after the second injection (P30). Mouse tibia and femurs were fixed overnight in 70% ethanol, embedded in OCT, cut 20 µm frozen sections. Images were obtained using a 25× objective (LSM510; Carl Zeiss).

### Western blot assay

Cells were lysed with RIPA lysis buffer (50 mM Tris-HCl (pH 7.4), 150 mM NaCl, 0.5% sodium deoxycholate, 1% Triton X-100, 0.1% SDS) containing protease and phosphatase inhibitor cocktails (Pierce). Cell and brain tissue lysates were centrifuged at 14,000×*g* for 20 min at 4 °C. Equivalent proteins as determined by BCA assay (Pierce Biotechnology) were resolved by SDS-PAGE and subjected to western blotting with appropriate primary antibodies. All acquired images were subjected to densitometric quantitation using NIH Image software.

### Mitochondrial fractions

Mitochondrial fractions were obtained as previously described^43^. Briefly, BMSCs and OBs (D14, differentiation from BMSCs) from WT and APP^−/−^ mice were suspended in .0.5 mM PMSF, pH 7.6) and homogenized by passing through a 27-gauge needle 8 times. After incubation on ice for 15 min, unbroken cells and nuclei were removed by centrifugation at 740 g for 10 min. The supernatant was centrifuged at 9000×*g* for 10 min to obtain the cytosolic fraction (supernatant). The pellet was subjected to two additional rounds of homogenization and differential centrifugation. The mitochondrial fraction was pelleted by the final centrifugation at 14,000×*g* for 20 min. Protein concentrations were measured by BCA kit from Pierce.

### Immunofluorescence staining and confocal imaging analyses

Cells plated on coverslips in 12-well plates and fixed with 4% paraformaldehyde for 15 min, permeabilized with 0.1% v/v Triton X-100/1xPBS (10 min), and blocked with 10% normal goat serum prior to incubation with primary antibodies overnight. Rabbit anti-ki67 (Millipore, 1:200), rabbit anti-Tom20 (Thermofisher, 1:1000), mouse anti-cytochrome C (BD, 1:500), rabbit anti-APP (Cell sig, 1:200), mouse anti-P62 (abcam, 1:500) and mouse anti-Tom20 (Santa Cruz, 1:100). Cells were then labeled with appropriate secondary antibody raised in goat (Invitrogen). Images were taken with the LSM800 (Zeiss) confocal microscope by using the 40×/0.5 EC Plan-Neofluar objective or the 63×/1.4 Oil Plan-Apochromat objective. Excitation and acquisition parameters were constrained across all paired comparisons. For fluorescent quantification, morphometric measurements of images were performed using Image-Pro Plus software (MediaCybernetics). Mitochondrial morphology quantification was conducted by the Mito-Morphology Macro^44^ through ImageJ software (National Institutes of Health) as previous described^43^. In brief, images of 30 cells from random view field of each group were first processed with a median filter to obtain isolated and equalized fluorescence, then individual mitochondria were analyzed for the lengths of major axes.

### Mitochondrial functional assay

Mitochondrial function was evaluated by oxygen consumption rate (OCR). It was measured in an X96 Extracellular Flux Analyzer with XF Cell Mito Stress Test Kit (Seahorse Biosciences) at 37 °C. BMSCs and OBs were plated on XF96 cell culture plate at 10 K per well and cultured for overnight with 80ul culture medium/well. Four wells without cells seeding from each plate were set as temperature and background control. For measurement, cells were gently rinsed with 100ul/well assay medium (XF Base medium (Seahorse Biosciences) with 2 mM Glutamine and 10 mM glucose), then put into 175ul/well assay medium and assayed. Three baseline OCR were calculated, followed by sequential injection of the ATP synthase inhibitor oligomycin, the mitochondrial uncoupler Carbonyl cyanide-4- (trifluoromethoxy) phenylhydrazone (FCCP) and the Complex I inhibitor rotenone. Two minutes OCR measurement were made at 3 min intervals with mixing and each condition was measured in each well.

### Discrimination of glycolytic versus OXPHOS ATP generation

To mimic OXPHOS suppression, oligomycin, a potent inhibitor of the mitochondrial ATP synthase, was used. To inhibit ATP production by the glycolytic pathway, 2-deoxyglucose (2DG) (the nonmetabolizable analog of glucose) was used. ATP level of BMSCs and OBs from WT and APP^−/−^ mice without any treatment or treated with 25 mM 2-DG or treated with 25 mM 2-DG plus 5 µm oligomycin for 45 min were measured with ATP Assay Kit (BioAssay Systems, Hayward, CA, USA) according to the manufacturer’s instructions. Briefly, ATP released from cells immediately reacted with the substrate d-luciferin to produce light in the presence of luciferase and read luminescence on a luminometer within 1 min after adding reconstituted reagent.

### Determination of ROS

The intracellular level of ROS is an important biomarker for oxidative stress and increased ROS level indicates increased oxidative stress generally. In this study, production of ROS was estimated with the fluorescent dye 2′7′-dichlorodihydrofluorescein diacetate (H_2_DCFDA; Thermofisher, Waltham, MA), a nonpolar compound that is converted by endogenous esterases to polar and membrane impermeable derivative H_2_DCF. H_2_DCF is nonfluorescent but in the presence of intracellular ROS is oxidized to fluorescent 2′,7′-dichlorofluorescein (DCF). For measurement, BMSCs and OBs from WT and APP^−/−^ mice were washed once with PBS and loaded with 20 µM H_2_DCFDA according to the manufacturer’s instructions. After cells were incubated at 37 °C for 30 min in the dark, the dye was removed, and cells were washed once with PBS and all of the samples were observed using confocal microscope immediately.

For measurement of mitochondrial O_2_^•‾^ production, BMSCs and OBs cultured from 2-M WT and APP^−/−^ mice were loaded with 5 μM MitoSOX (Invitrogen) in PBS containing 1 g/l glucose for 10 min at 37 °C incubator, washed with PBS for two times, and the fluorescence emission at 595 nm under 510 nm excitation was recorded using a microplate reader as previously described^45^.

### Cell death assay

The caspase family plays a key role in the molecular mechanisms of inducing cell death and is ultimately involved in apoptosis, among which caspase 3, also called “death protease”, makes cell apoptosis inevitably once activated^46^. So we did immunofluorescence staining of cleaved-caspase 3(rabbit, CST, #9664S) to observe positive cells’ percentage in both BMSCs and OBs from WT and APP^−/−^ mice.

### Transfestion of OBs with Mito-RFP and Sec61β-GFP

WT and APP^−/−^ OBs were and were plated at 1 × 10^4^/well onto 12-wells coverslips and grown in DMEM containing 10% (vol/vol) FBS, and 50 units/ml penicillin and streptomycin plus 10 mM β-glycerophosphate and 50 μM l-ascorbic acid-2- phosphate. At D10, cells were transfected with Mito-RFP and Sec61β-GFP by Lipofectamine 3000 (Invitrogen), 72 h after transfection, cells were fixed and imaged by LSM800 (Zeiss) confocal microscope with the 63×/1.4 Oil Plan-Apochromat objective.

### MC3T3 cell culture and generation of APP-KO MC3T3 cell line

MC3T3-E1 cells were grown in DMEM containing 10% (vol/vol) FBS, and 50 units/ml penicillin and streptomycin. To differentiate pre-osteobalstic MC3T3 cells to mature osteoblastic MC3T3 cells, cells were induced by the addition of 50 ng/ml ascorbic acid and 10 mM β-glycerophosphate to standard growth medium for 7 days. All MC3T3 Cells were plated at 1 × 10^4^/well onto 12-wells coverslips the day before transfection with LC3-GFP/Mito-RFP/ CAG-LC3-mCherry-GFP plasmids.

For generation of APP-KO MC3T3 cell line, PX330 vector was purchased from Addgene. The puromycin resistant gene was replaced by GFP. Guidance RNA CACCGTGTATGCTGCCCGTTGGCCG for App was inserted into Bbsl restriction site and verified by sequencing. The PX330 plasmid was then transfected to MC3T3 cells and GFP positive cells were sorted by fluorescence-associated cell sorting(FACS) and cultured in 96-well plate with one cell per well. 4–5 days later, the cells were passed to 12-well plate, and the App-KO clones were identified by western blot and PCR-based genomic DNA sequencing.

### RNA isolation and real-time PCR

Total RNAs were isolated from primary cultured BMSCs, OBs (D14 culture) by Trizol extraction (Invitrogen, Carlsbad, CA, USA). Quantitative PCR was performed with Quantitect SYBR Green PCR Kit (Qiagen) according to the manufacturers’ instructions and a Real-Time PCR System with analytical software (Opticon Monitor 3). The following primers were used: P62, 5′-ACATACGCAGAACAGAGTTACGAAGG-3′ and 5′-CATTCCAGTCATCTTGTCCGTAGGC-3′; GAPDH primers (5′-AAGGTCATCCCAGAGCTGAA -3′ and 5′-CTGCTTCACCACCTTCTTGA-3′ were used for normalization.

### NAC treatments

For in vitro treatment, D1-OB cultures (differentiated from BMSCs) were treated with NAC (Sigma Aldrich, 1 mM, overnight) or veh. control, then changed to OB differentiation medium. At D14, mitochondrial function assay and ALP staining were carried out.

For in vivo treatment, WT and APP^−/−^ mice were divided into two groups separately: (1) one group of mice (*n* = 5, male) received drinking water, and (2) another group of mice (*n* = 5, male) received drinking water containing NAC (2 mg/kg/day) for a period of 3-M. NAC in water (0.001%, pH adjusted to 7.2 by NaOH) was prepared. Water was changed every other day for 3-M. Approximately 4–5 ml of water was consumed by each mouse per day, which gives a cumulative dose ~2 mg/kg per mouse per day. This NAC dose was chosen based on previous publications^15,47^^.^ The NAC treatment was started at age of 1–2 M old. After 3-M of the treatment, mice were euthanized, and their femur bone and sera samples were collected for histomorphometric and μCT analyses, and for ELISA analysis of PYD and osteocalcin levels, respectively.

### Statistical analysis

All assays were repeated in three independent experiments. All data were presented as mean ± SEM. Significance was determined by Student’s *t*-test for pairwise comparison and one-way ANOVA with Bonferroni post-test for multiple comparisons using GraphPad Prism 6.0 software (Graphpad Software, Inc., La Jolla, CA, USA). The significance level was set at *p* < 0.05.

## Electronic supplementary material

Supplemental figures and legends
