# Supplementary material for: APP promotes osteoblast survival and bone formation by regulating mitochondrial function and preventing oxidative stress
Source: Cell Death Dis. 2018 Oct 22;9(11):1077. doi: 10.1038/s41419-018-1123-7 (PMC6197195; doi:10.1038/s41419-018-1123-7)
Supplement: Supplementary file 1 — Supplemental figures and legends [file 41419_2018_1123_MOESM1_ESM.docx]

Supplemental Figures


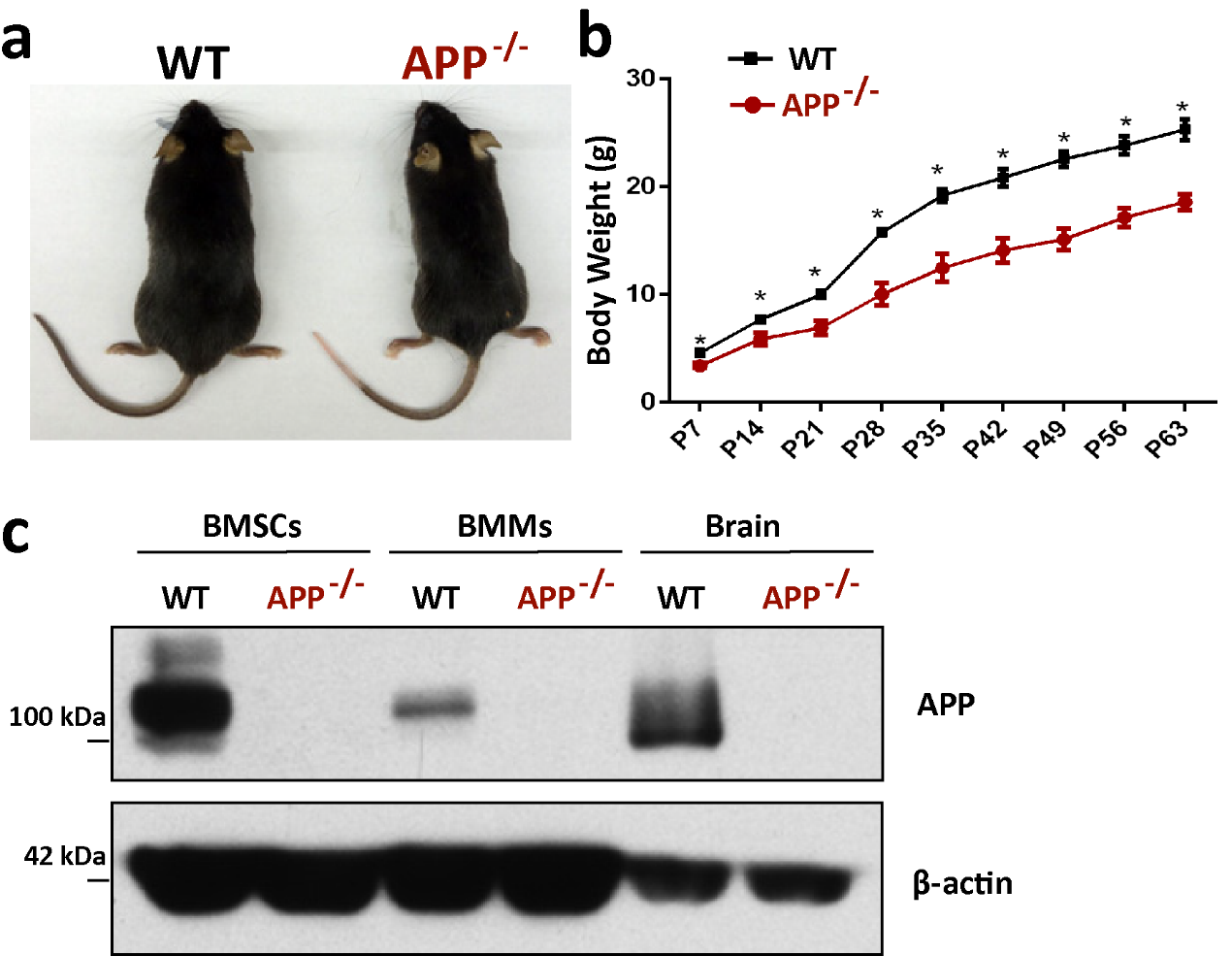


Fig. S1 Reduced body weight in APP^-/-^ mice. (a) Images of WT and APP^-/-^mice (male) at age of 2-M old. (b) Reduced body weights in APP^-/-^ mice, compared with same age same gender WT control mice. (c) Western blot analysis of APP protein levels in lysates of BMSCs, BMMs, and brain derived from WT and APP^-/-^ mice (2-M old). Antibody (APP, #2452, Cell Signaling) was used.

**
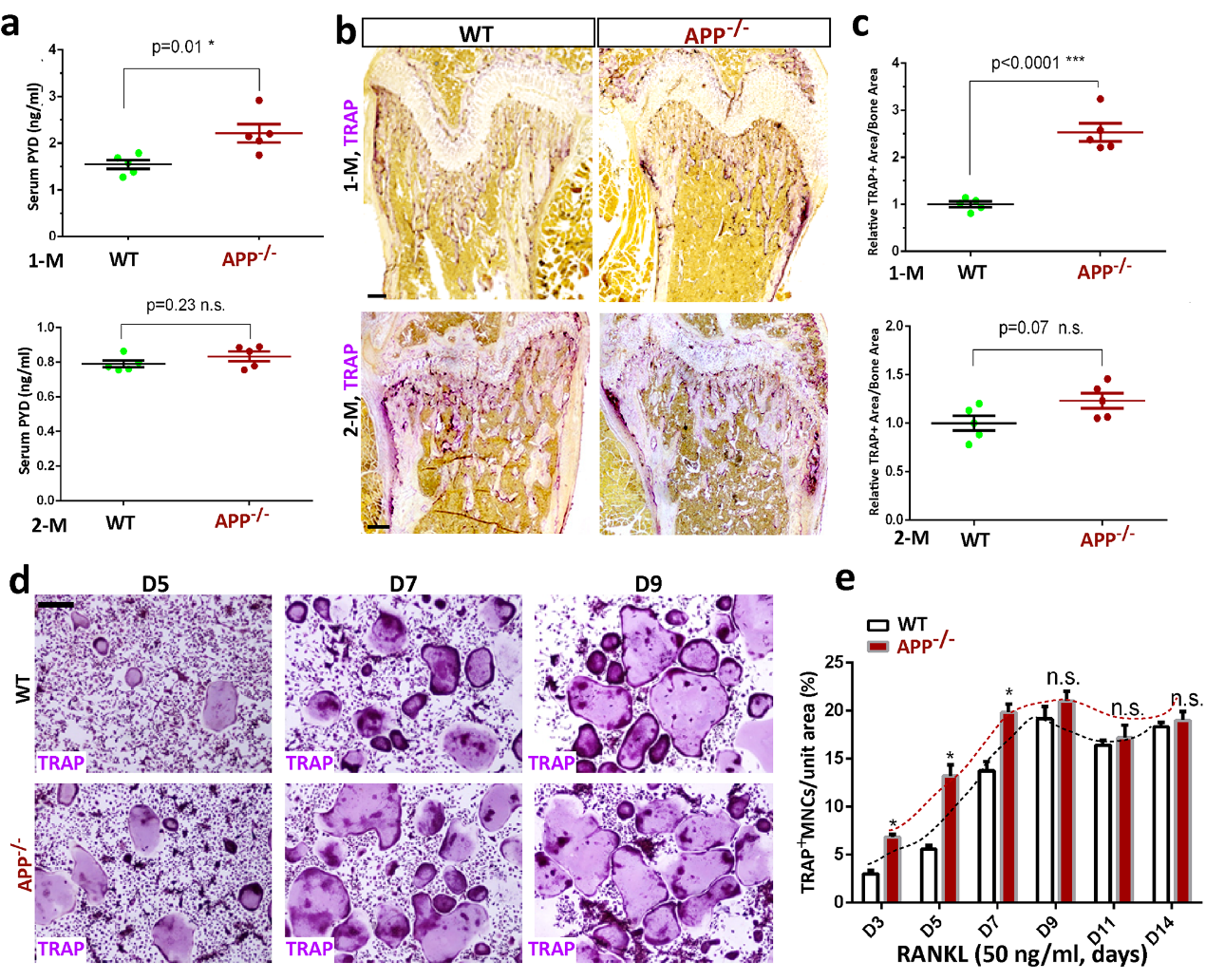
**

**Fig. S2 Transient increase of bone resorption and OCs in neonatal APP^-/-^ mice and in APP^-/-^ BMM to OC cultures. (a)** Measurement of serum levels of PYD from 1- and 2-M old WT and APP^-/-^ mice. **(b-c)** TRAP staining analysis of femur sections from 1- and 2-M old WT and APP^-/-^ mice. Representative images of TRAP staining were shown in (b), scale bar, 100 µm. The quantitative analysis of TRAP^+^ cells per unit bone surface (BS) was carried out in trabecular bones of femurs and showed in (c). In (a and c), the values of mean ± SEM from 5 different animals (males) per genotype per age group were shown. **(d-e)** In vitro OC genesis of BMMs derived from WT and APP^-/-^ mice (1-M old). OCs were generated from purified BMMs (5 x 10^4^) cultured in the presence of RANKL (100 ng/ml) and M-CSF (1%) for indicated days. Representative images of TRAP staining were shown in (d), scale bar, 200 µm. Quantitative analyses of the average TRAP positive multi-nuclei cell (MNC) density [TRAP^+^ MNCs (>3 nuclei per cell) per unit area] were presented in (e). The values of mean ± SEM from 3 separate cultures were shown. *, *p* < 0.05, ***, *p* < 0.0001.


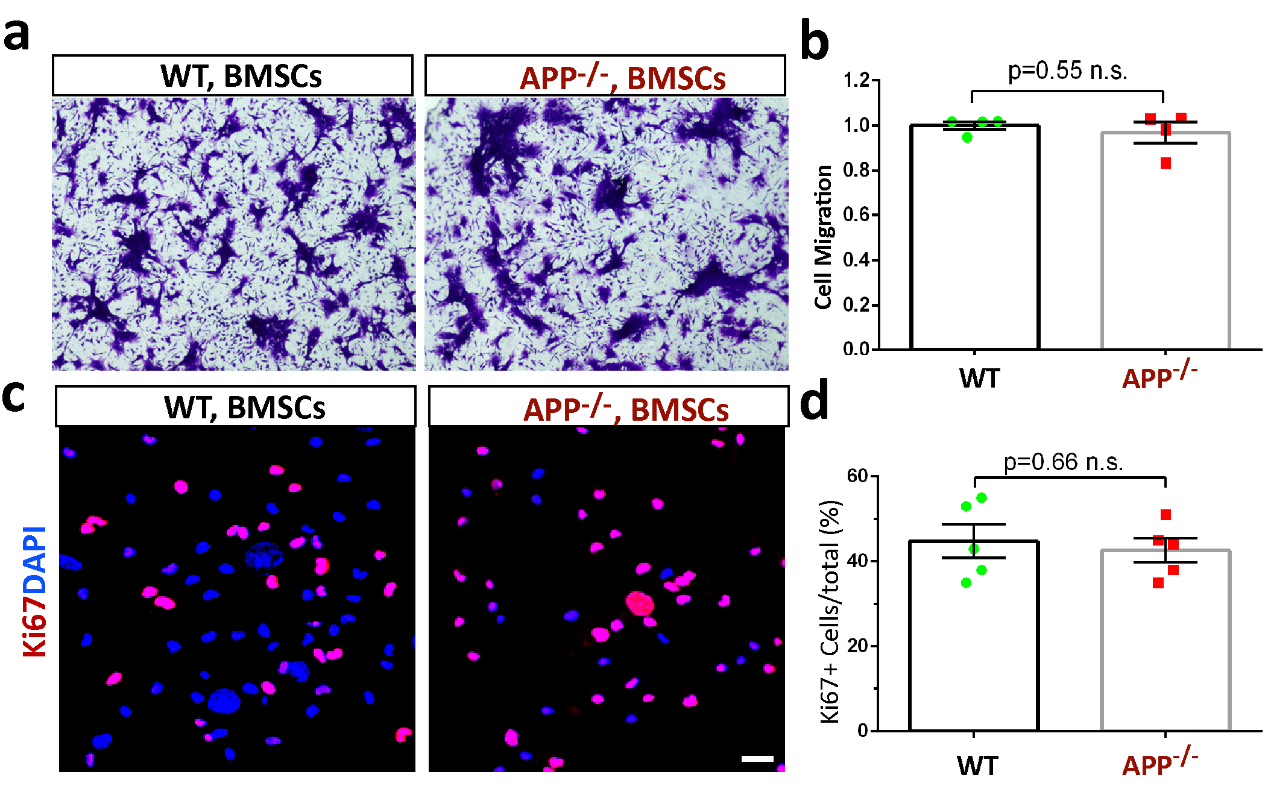


Fig. S3 No change of BMSC migration and proliferation in APP^-/-^ BMSC cultures. (a-b) Transwell cell migration assay showed little to no change of APP^-/-^ BMSC migratory potentials. (a) Microscopic image of crystal violet staining; **(b)** The quantification analysis (mean ± SEM; n >500 cells from 4 separate cultures). (c-d) Immunostaining analysis of Ki67 (a marker for cell proliferation) showed little to no change of APP^-/-^ BMSC proliferation. c, Representative images, scale bar, 20µm. d, Quantification analysis (mean ± SEM, n=5 mice per genotype).


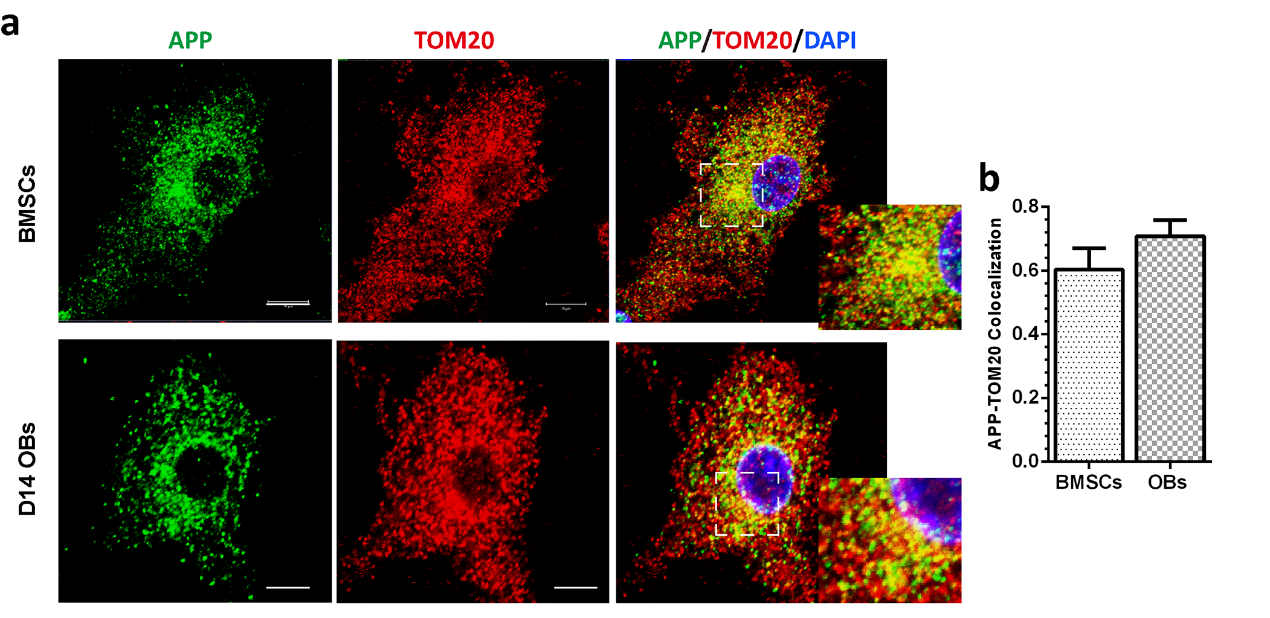


Fig. S4 APP distribution in mitochondria in primary cultured WT BMSCs and D14-OBs. (a) Representative images of immunostaining analysis using indicated antibodies. Scale bar: 10μm. (b) Quantification of APP co-localization with mitochondria (Tom20) (mean ± SEM; n = 30 cells from 3 separate cultures).


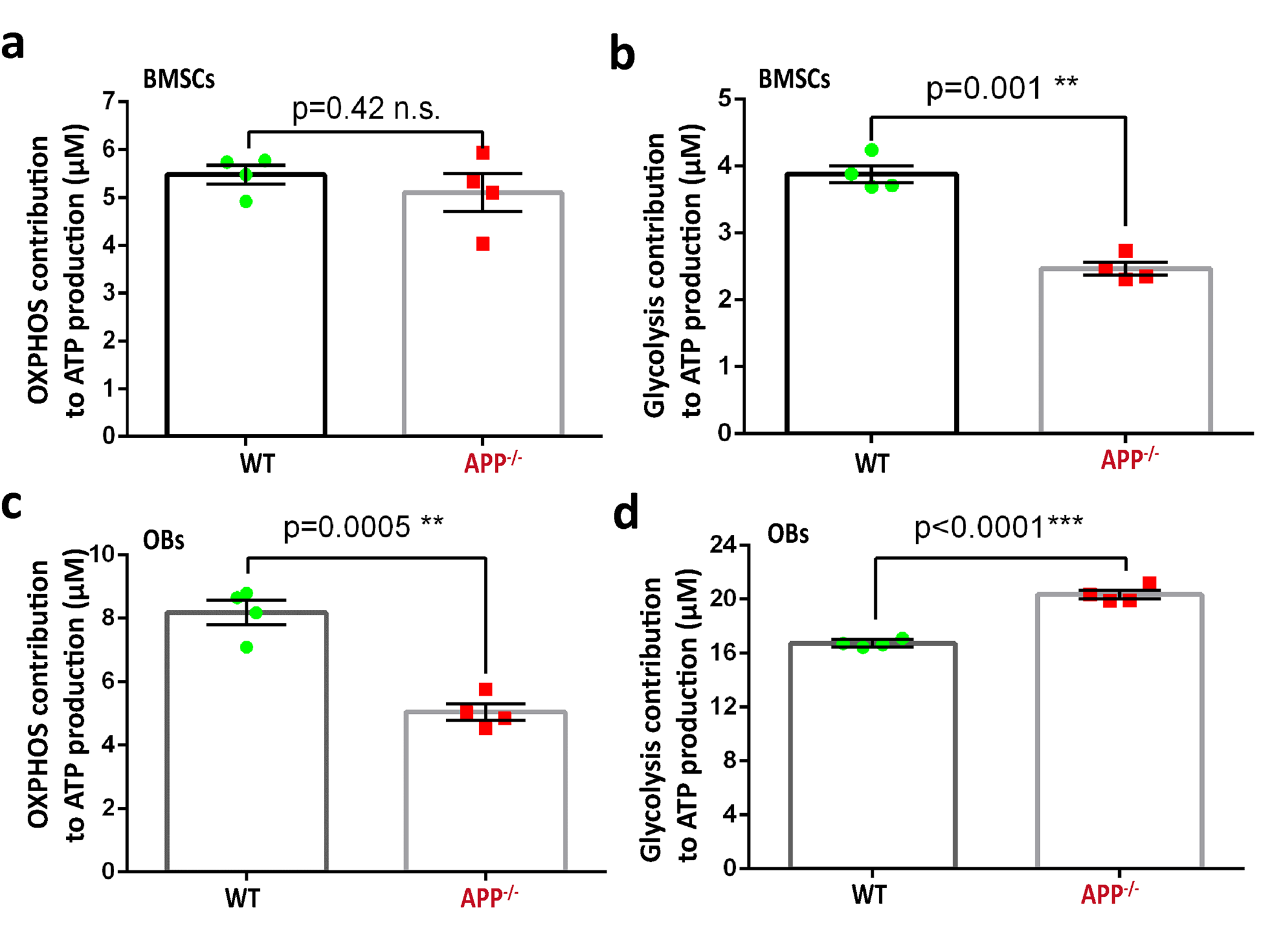


**Fig. S5** **Glycolysis-dependent and mitochondrial oxidative phosphorylation (OXPHO)-dependent ATP production in WT and APP^-/-^ BMSCs and OBs.** (**a-d**) WT and APP^-/-^ BMSCs and OBs were plated on 96-well plates in the presence or absence of 2-Deoxy-D-glucose (2-DG) or 2-DG with oligomycin for 45 min. After incubation, their intracellular ATP levels were measured by Bioassay ATP Kit. ATP level by glycolytic pathway, but not OXPHO-pathway, was reduced in APP^-/-^ BMSCs, the quantification were shown in (a-b). OXPHOS-dependent ATP production was reduced and the glycolytic dependent ATP level was increased in APP^-/-^ OBs, data were shown in (c-d). All data were shown as mean **±** SEM, n=4 mice/genotype. *, p < 0.05; **, p < 0.01; ***, p< 0.0001.


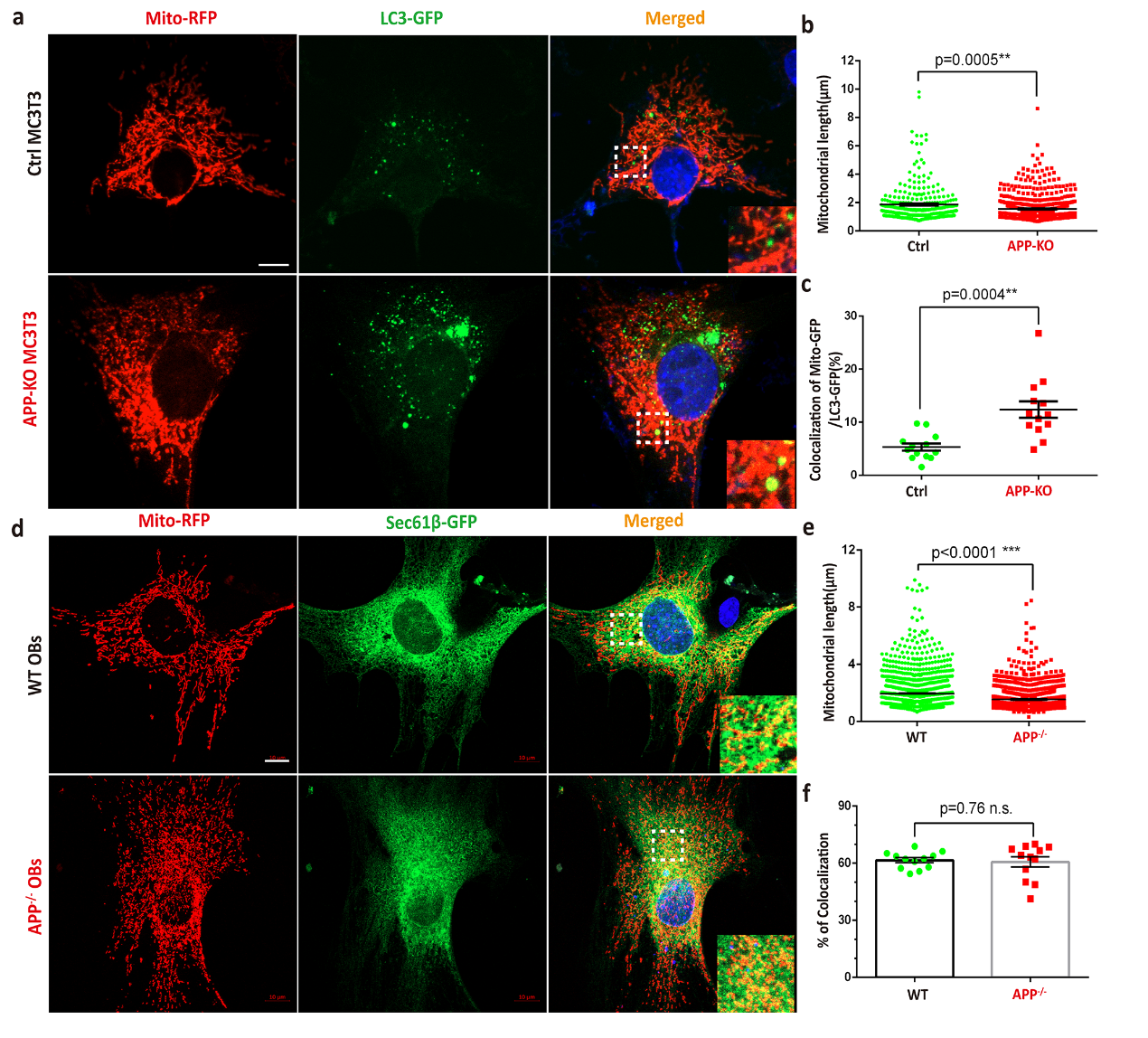


**Fig. S6** **Increased mitophage in differentiated APP-KO MC3T3 cells and No change of ER-mitochondria contact sites in APP^-/-^ OBs.** (**a-c**) Control and APP-KO MC3T3 Cells were cultured in growth and differentiation medium for 7-days. Then those cells were co-transfected with plasmids encoding LC3-GFP and Mito-RFP. 3 days after transfection, Cells were fixed and subjected to fluorescence imaging analysis. Representative images shown in (a), Scale bar, 10µm; **(b-c)** the quantification analysis of mitochondria length (mean ± SEM; n>1000 mitochondrial from 13 cells from 3 separate cultures) and colocalization of Mito-GFP and LC3-GFP over LC3-GFP (mean ± SEM, n =13 from 3-different cultures), **, P < 0.01. (**d-f**) Transient transfection of plasmids encoding Sec61 β-GFP (an ER marker) and mito-RFP (a mitochondrial marker) in WT and APP^-/-^ OBs.  Representative images shown in (d), Scale bar, 10µm; **(e-f)** the quantification analysis of mitochondria length (mean ± SEM; n>1000 mitochondrial from 12 cells from 3 separate cultures) and colocalization of Mito-GFP and Sec61β-GFP over Mito-RFP (mean ± SEM, n =12 from 3-different cultures), ***, P < 0.0001.
